# Insights into *S. aureus*-Induced Bone Deformation in a Mouse Model of Chronic Osteomyelitis Using Fluorescence and Raman Imaging

**DOI:** 10.3390/ijms24119762

**Published:** 2023-06-05

**Authors:** Shibarjun Mandal, Astrid Tannert, Christina Ebert, Rustam R. Guliev, Yvonne Ozegowski, Lina Carvalho, Britt Wildemann, Simone Eiserloh, Sina M. Coldewey, Bettina Löffler, Luís Bastião Silva, Verena Hoerr, Lorena Tuchscherr, Ute Neugebauer

**Affiliations:** 1 Leibniz Institute of Photonic Technology (Member of Leibniz Health Technologies, Member of the Leibniz Centre for Photonics in Infection Research, LPI), 07745 Jena, Germany; 2 Center for Sepsis Control and Care, Jena University Hospital, 07747 Jena, Germanylorena.tuchscherrdehauschopp@med.uni-jena.de (L.T.); 3 Institute for Medical Microbiology, Jena University Hospital, 07747 Jena, Germany; 4 Institute of Anatomical and Molecular Pathology, Faculty of Medicine, University of Coimbra, 3004-504 Coimbra, Portugal; 5 Experimental Trauma Surgery, Jena University Hospital, 07747 Jena, Germany; 6 Department of Anaesthesiology and Intensive Care Medicine, Jena University Hospital, 07747 Jena, Germany; 7 BMD Software, PCI-Creative Science Park, 3830-352 Ílhavo, Portugal; bastiao@bmd-software.com; 8 Heart Center Bonn, Department of Internal Medicine II, University Hospital Bonn, 53127 Bonn, Germany; 9 Institute of Physical Chemistry and Abbe Center of Photonics, Friedrich Schiller University Jena, 07743 Jena, Germany

**Keywords:** chronic bone infection, *Staphylococcus aureus*, mouse model of osteomyelitis, *staphylococcal abscess communities (SACs)*, label-free imaging, intracellular bacteria, second harmonic generation, inflamed tissue, X-ray, host response, Immunofluorescence imaging, Raman imaging, spectral unmixing, small colony variant (SCV), fibroblast, pelvis

## Abstract

Osteomyelitis is an infection of the bone that is often difficult to treat and causes a significant healthcare burden. *Staphylococcus aureus* is the most common pathogen causing osteomyelitis. Osteomyelitis mouse models have been established to gain further insights into the pathogenesis and host response. Here, we use an established *S. aureus* hematogenous osteomyelitis mouse model to investigate morphological tissue changes and bacterial localization in chronic osteomyelitis with a focus on the pelvis. X-ray imaging was performed to follow the disease progression. Six weeks post infection, when osteomyelitis had manifested itself with a macroscopically visible bone deformation in the pelvis, we used two orthogonal methods, namely fluorescence imaging and label-free Raman spectroscopy, to characterise tissue changes on a microscopic scale and to localise bacteria in different tissue regions. Hematoxylin and eosin as well as Gram staining were performed as a reference method. We could detect all signs of a chronically florid tissue infection with osseous and soft tissue changes as well as with different inflammatory infiltrate patterns. Large lesions dominated in the investigated tissue samples. Bacteria were found to form abscesses and were distributed in high numbers in the lesion, where they could occasionally also be detected intracellularly. In addition, bacteria were found in lower numbers in surrounding muscle tissue and even in lower numbers in trabecular bone tissue. The Raman spectroscopic imaging revealed a metabolic state of the bacteria with reduced activity in agreement with small cell variants found in other studies. In conclusion, we present novel optical methods to characterise bone infections, including inflammatory host tissue reactions and bacterial adaptation.

## 1. Introduction

Osteomyelitis is an infection of the bone with pyogenic organisms, including different bacteria and mycobacteria [1]. An occurrence of 16.7 cases per 100,000 individuals in the adult population in Germany was reported for the year 2018 with an increasing trend [2]. Similar numbers and trends were obtained from studies in the United States [3]. Based on the etiology, osteomyelitis can be divided into hematogenous osteomyelitis, osteomyelitis due to a contiguous focus of infection or osteomyelitis due to vascular insufficiency [4,5]. Osteomyelitis can affect any bone in the body, including those in the pelvis. The incidence of osteomyelitis in the pelvis is relatively rare, accounting for only a small fraction of all cases [6], in adults often associated with pressure ulcera [7]. Symptoms of osteomyelitis may include local signs of infection, such as redness, swelling, warmth, and pain at the site of infection (for pelvis osteomyelitis, it can manifest as groin pain), as well as general symptoms, such as fever or chills. Acute osteomyelitis presents within approximately two weeks after a bone infection, characterised by inflammatory bone changes. Chronic osteomyelitis develops after several weeks to years of persistent infection and is characterised by bone destruction and the formation of sequestra. Bone infections, especially chronic ones, are often difficult to treat and result in long therapies, including surgery and antibiotic treatment [4,8]. Reasons for this include adaptation strategies of the pathogens that help them to evade immune responses and treatment [8,9,10,11]. *Staphylococcus aureus* (*S. aureus*) is the most common bacterium causing osteomyelitis [8,12,13]. It was found to use various strategies, such as the formation of a biofilm and abscesses in soft tissue or bone marrow, colonization of the osteocyte-lacuno canalicular networks, intracellular persistence, and the formation of small colony variants with reduced metabolism [14].

A diagnosis of osteomyelitis usually starts with serum and synovial fluid tests to confirm inflammation and peripheral blood cultures to hopefully identify the pathogen during culture [8]. Diagnosis is followed by site-directed advanced imaging, such as magnetic resonance imaging, plain radiography, CT scan, and nuclear medicine studies (e.g., positron emission tomography (PET) or technetium-99m bone scans), to visualise the location and extent of the osteomyelitis [15]. Despite all imaging advances, an additional tissue biopsy for bone histology remains the diagnostic standard [7] and is often necessary to reveal the cause of the infection and to decide on an appropriate therapy [8,16].

Crucial for the advancement of the understanding of the pathophysiology of osteomyelitis was the use of animal models [17,18]. Together with a powerful tissue analysis, including histopathology and imaging on a micrometer and submicrometer scale, the host’s response to the infection as well as resulting changes in bone morphology could be visualised [19,20,21]. Several histopathological staining methods are established to stain the bone structure [22]. Fluorescence microscopy allows for the visualization of selectively stained structural features using cells or features in fixed tissue slices, enabling diffraction-limited spatial resolution, a high contrast, and the creation of 3D images by examining different optical planes [23]. An emerging optical analysis method that does not require any staining or labelling procedure to provide information about the overall molecular composition and three-dimensional structure is Raman spectroscopic imaging. This vibrational spectroscopic technique has the potential to be used as a non-invasive in vivo imaging and diagnostic tool as was already previously demonstrated in the context of cancer detection [24,25]. For a bone analysis, it was previously explored not only to characterise the bone quality [26,27,28,29], but also to characterise changes in the (mineralised) bone due to an infection [30] and arthritis [31] and to identify bacteria in bone grafts [32]. However, the potential of Raman spectroscopy has not yet been explored to characterise tissue changes during osteomyelitis.

In this study, we applied confocal laser scanning microscopy as well as Raman spectroscopic imaging to extensively characterise *S. aureus*-induced pelvis deformation during chronic hematogenous osteomyelitis on a microscopic scale. Bacteria were localised in all regions of the bone and osseous, and soft tissue changes as well as inflammatory lesions were characterised with the different imaging modalities and put into relation to classical histopathological staining, such as hematoxylin and eosin staining as well as Gram staining. Bacterial abscess communities and lesions were also found in the macroscopically not-deformed side of the pelvis.

## 2. Results and Discussion

### 2.1. Infection Caused Phenotypic Changes

From two days after infection, the mouse showed slowed movement and started limping, especially in the hind limps. During the first four days post-infection (p.i.), the mouse lost about 20% of its weight (see Supplementary Graph S1). After seven days p.i., the mouse began to regain weight gradually and reached 95% of its initial weight by the end of the observation period (week six). At the end of week two and during week three p.i., the behaviour of the mouse became almost normal. However, in week four, the mouse developed toddling movement. Starting from the end of week four p.i., a swelling became visible at the rump.

### 2.2. X-ray Imaging Revealed Macroscopic Signs of Osteomyelitis

**Figure 1:**
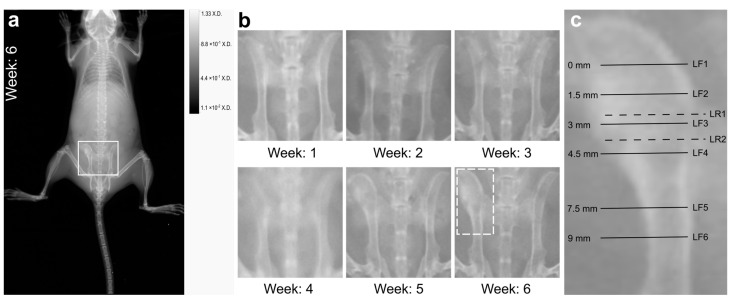
X-ray imaging reveals macroscopic bone changes during the course of infection. (**a**) Whole body scan of the mouse with chronic hematogenous osteomyelitis 6 weeks after infection. The white rectangle highlights the region of the pelvis shown in panel (**b**). (**b**) Enlarged region of the pelvis from the weekly X-ray images during the course of infection. The white rectangle at week 6 highlights the deformed left pelvis used for cryo-sectioning. (**c**) Left pelvis with labelled cryo-sections from the ilium discussed further: LF1, LF2, LF3, and LF4, are each separated by a distance of 1.5 mm. LF5 is positioned 3 mm away from LF4, while LF6 is 1.5 mm away from LF5. All of these sections were used for immunofluorescence staining. LR1 and LR2 are cryo-sections used for Raman spectroscopic imaging. LR1 is 100 µm apart from LF3 (towards LF2), LR2 is 400 µm apart from LF3 (towards LF4).

X-ray imaging was done weekly during the six week course of infection to detect any changes in bone structure. In the first week after infection, no pathological transformation could be observed. However, as visible in Figure 1a, severe bone deformations with thickening of the ilium were clearly visible in the left pelvis six weeks after infection. Figure 1b highlights the changes occurring in the pelvis region during progression and manifestation of the infection. No major alterations were seen in the 2D X-ray image of the right pelvis.

### 2.3. On Microscopic Scale, Severe Lesions and Bacterial Abscesses Were Visible

**Figure 2:**
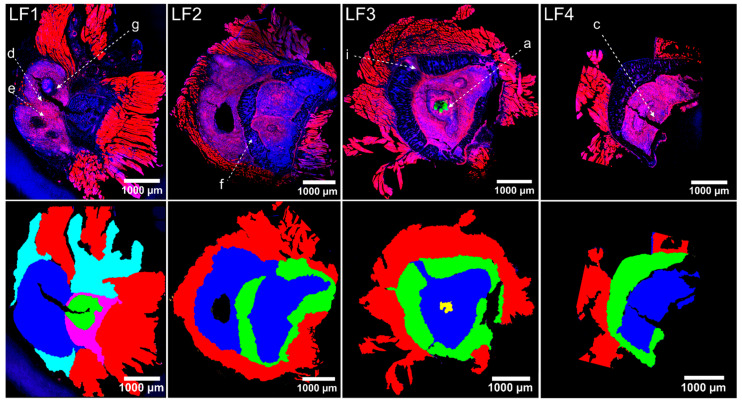
Confocal images of immunofluorescence-labelled pelvis cryo-sections LF1, LF2, LF3, and LF4 (as indicated in Figure 1c). (**Top row**): Immunofluorescence images. Fluorescence staining highlights cell nuclei (DAPI, blue), actin-cytoskeleton (I555-Phalloidin, red), *S. aureus* (specific antibody AF488: green). The pink colour arises here from the overlay of nuclei and actin-cytoskeleton. Alphabetically labelled regions (a, c–g, i) refer to panel labels of enlarged views in Figure 3. (**Bottom row**): Colour-coded slices according to structures. Trabecular bone (green) and lesions (blue) are visible in all slices. Muscle tissue (red) is still attached to the periosteum. Staphylococcal abscess communities (SACs) are visible in section LF3 (yellow).

Tissue samples of the left and the right pelvis six weeks after infection were characterised by microscopic and microspectroscopic methods. Overview CLSM images of immunofluorescently stained left pelvis tissue sections are shown in Figure 2 (1st row). The sections were each around 1.5 mm apart (i.e., spanning a distance of around 4.5 mm between section LF1 and section LF4 (as indicated in Figure 1c)). In each of the displayed tissue sections, three tissue types were present: trabecular bone tissue, muscle tissue, and a lesion (see also respective colour-coding in Figure 2, second row). Moreover, section LF1 exhibited a region rich in chondrocytes and a highly vascularised area. In addition, section LF3 showed several abscesses filled with *S. aureus,* identifiable by the intense green staining with *S. aureus* antibodies in the core of the lesion (Figure 2). These abscesses and other bacterial localizations will be discussed in more detail in Section 2.5.

**Figure 3:**
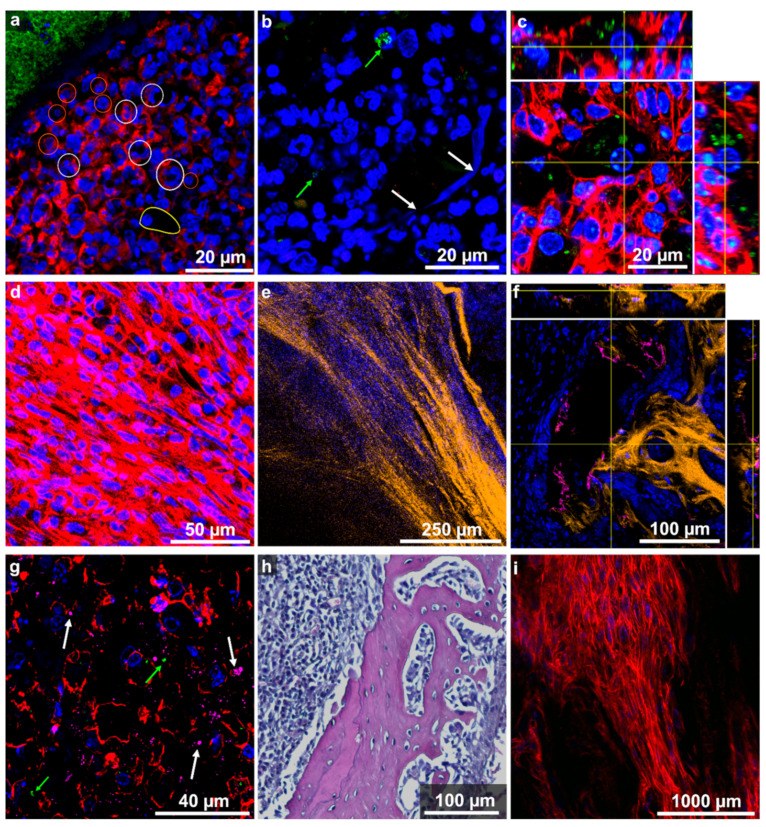
Detailed images of host tissue structures in infected pelvis. Colour-code of channels is given below. (**a**) Accumulation of immune cells around the SAC (green-stained *S. aureus*), e.g., neutrophils (white circle), lymphocytes (orange circle), macrophages (in contour) assigned based on the nuclear shape (DAPI) and size (immunofluorescence, LF3) (**b**) Presence of NETs (white arrows) in close vicinity to *S. aureus* infiltrates (green arrows) in the lesion (immunofluorescence, RF2). (**c**) Orthogonal view of a foamy macrophage with bacteria phagocytosed (immunofluorescence, LF4). (**d**) Accumulation of fibroblast (immunofluorescence, LF1). (**e**) Deposition of collagen observed using SHG microscopy in inflamed tissue next to nuclei of fibroblasts (immunofluorescence, LF1). (**f**) Ortho-view showing osteocalcin deposits (pink) at the edges of mineralised, collagen-rich (orange) bone (immunofluorescence and SHG microscopy, LF2). (**g**) Immunofluorescence image of osteocalcin-rich (pink) area in soft tissue. Single bacteria were identified (green arrows) in addition to some cells with high osteocalcin production (white arrows) (LF1). (**h**) H&E staining reveals spindle-like cells in the trabecular spaces of infected bone tissue (RH1). (**i**) Broken trabecular structure infiltrated with the lesion (immunofluorescence LF3). Channel colours for the immunofluorescence images mark the following features (and used fluorophore): blue: nuclei (DAPI or SYTOX green), red: actin-cytoskeleton (I555-Phalloidin), green: *S. aureus* (DY405 or AF488), pink: osteocalcin (Dy650), orange: collagen (SHG). Note that for clarity not all channels were shown in all panels.

The muscle fibers attached to the periosteum of the bone were characterised by strong actin staining (Figure 2, 1st row). Trabecular bone tissue showed a typical porous network structure with the blue-stained nuclei of the cells in the trabecular spaces and a surrounding bone matrix which appeared dark in the fluorescence images. The shape and size of trabecular bone tissue varied between slices LF1 and LF4. While it formed a well-defined region with homogenous trabecular structure in section LF1, large lesions were visible in the trabecular bone in slices LF2–LF4. The lesions were characterised by a relatively high density of nuclei and intense phalloidin staining of the actin-cytoskeleton, which let these regions appear pink in Figure 2. The lesions made up a significant portion of the bone tissue across all slices. It was found either surrounded by trabecular bone (slices LF2, LF3, LF4) or outside of the trabecular bone (slices LF1, LF2). In slice LF2 and LF3, the lesions seemed to break through the regular trabecular structure and infiltrated it (Figure 2, LF3 is enlarged in Figure 3i). The large size of the lesion may have caused the tissue deformation that was observable in X-ray imaging (Figure 1) and during the macroscopic examination after the bone was prepared.

Interestingly, tissue slices of the right pelvis show very similar features on a microscopic scale despite the absence of visible bone deformations in the corresponding X-ray image (Figure 1). Areas of lesions containing multiple abscesses filled with *S. aureus* were clearly visible in fluorescence, H&E, and Gram-stained images displayed in Supplementary Figure S1. In another tissue section of the right pelvis (R2), an extensive lesion was present, but no SACs were present (Supplementary Figure S1). Bacteria were scattered as individual bacteria or as small clusters throughout the lesion.

From the tissue slice, the rough bone area was estimated by adding up the area of the trabecular bone and lesion and—if present—chondrocyte-rich tissue, highly vascularised regions, and bone-marrow structures, but excluding the muscle tissue (Supplementary Table S2). In average, tissue slices of the left pelvis were larger (8.0 ± 2.8 mm^2^) than tissue slices of the right pelvis (4.9 ± 0.8 mm^2^). This agrees with the observed macroscopic deformation and thicker bone size of the left pelvis. The percentage of bone tissue with lesions varied considerably between the different slices and was highest in LF2. On average, the trabecular bone made up 33.6 ± 20.6% of the left pelvis sections and, similarly, 30.1 ± 4.5% of the right pelvis sections (see Supplementary Table S2).

### 2.4. Host Tissue Feature Analysis Indicated the Presence of a Chronically Florid Osteomyelitis

In enlarged images, Figure 3 highlights typical host tissue changes caused by the infection. Very similar features of tissue modifications were found in the left and right pelvis. As mentioned before and will be discussed in Section 2.5, the presence of staphylococcal abscess communities (SACs) is a sign of an active infection. In close vicinity to the SACs, several immune cells can be identified based on the shape of their nucleus. In particular, neutrophils with their multi-lobed nuclei, different lymphocytes with their round askew nuclei, and large macrophages are highlighted in Figure 3a and mark an inflammatory infiltrate pattern. In a few areas of the inflammatory tissue in the vicinity to bacteria, we observed extracellular trail-like features with intense nucleic acid staining (Figure 3b). These can be assigned to the DNA of neutrophils released as neutrophil extracellular traps (NETs) and mark the migration path of neutrophils in the fight against the invading bacteria [33,34]. Other signs of an active immune response are accumulations of foamy macrophages with phagocytosed *S. aureus* (Figure 3c).

The region around the SACs contains not only a lot of immune cells, but it is also filled with an accumulation of inflammatory tissue mainly consisting of actin-rich fibroblastic cells (Figure 3d). Those cells were found to be the major component of the extensive lesion. Together with the collagen deposition (visualized by SHG microscopy, Figure 3e), these are clear signs of fibrosis and indicate a proliferative state in these areas [35].

At the edges of collagen-rich mineralised bone areas, we observed osteocalcin depositions (Figure 3f and Supplementary Figure S2). Osteocalcin staining was also observed in some confined tissue areas, as shown in Figure 3g, and localised in certain regions of the lesion (Supplementary Figure S3, LF1). The overview images in Supplementary Figure S3 show that in all tissue slices LF1 to LF4 and also in two slices further apart (LF5 and LF6), regions with high osteocalcin staining were found. High osteocalcin staining marks osteoblastic activity and can be interpreted as a sign for reactive bone formation and osteoneogenesis [19]. The location of the osteocalcin staining in the pelvic bone slices indicates a new bone formation as part of the physiological remodelling and also as a repair process of the lesions.

In bone slices, which were dominated by lesion tissue and also cells in the trabecular spaces, appeared to be rather spindle-like (Figure 3h) and very different from the typical round precursor cells found in healthy bone marrow of the pelvis from an uninfected, healthy mouse (Supplementary Figure S4 HRH). This indicates the presence of fibroblasts, even in the trabecular spaces of the osteomyelitis bones. In a tissue slice far away from the SAC (in LF5, 4,5 mm away from LF3), bone marrow cells appeared to be round and healthy. Interestingly, in LF6, a tissue slice already 6 mm away from LF3, cells started to appear rather spindle-like again, which might point to another infected region, maybe one similar to the one observed in the right pelvis where a deformation was not yet observed.

A highly vascularised region was found, especially in slice LF1 (Supplementary Figure S5). As only one time point of the tissue analysis is present, it cannot be clearly stated that those are newly formed vessels.

In summary, on a microscopic level, typical signs of chronic osteomyelitis were observed. These are in particular severe osseous changes with bone neogenesis, soft tissue changes with fibrosis and granulation tissue (fibroblasts and infiltrated inflammatory cells), and inflammatory infiltrate patterns with infiltration of macrophages, lymphocytes, and neutrophilic granulocytes. In addition, features of acute osteomyelitis, such as pathogen phagocytosis and NETosis, can be seen. Using the Histopathological Osteomyelitis Evaluation Score (HOES) defined by Tiemann and coworkers [19], we can classify a chronically florid osteomyelitis.

Surprisingly, we can find very similar features on a microscopic level in the left and the right pelvis. Using HOES, a chronically florid osteomyelitis would be diagnosed in both tissue sides of the pelvis. Together with the tissue thickening observed in the left pelvis, it could be concluded that osteomyelitis-induced tissue remodelling is more advanced on the left side.

### 2.5. Localization and Quantification of S. aureus Using Fluorescence Imaging and Gram Staining

The presence of bacteria was observed in all investigated tissue sections of the left and the right pelvis as visualised by immunofluorescence using a specific *S. aureus* antibody and by Gram staining. Using high-resolution fluorescence imaging, we were able to detect bacteria in several µm depths of the tissue sections and with a high signal-to-noise ratio. The excellent spatial resolution obtained with the fluorescence images allowed the localization of individual bacteria as well as a semi-quantitative analysis of the presence of bacteria in the different tissue regions (as will be described below).

**Figure 4:**
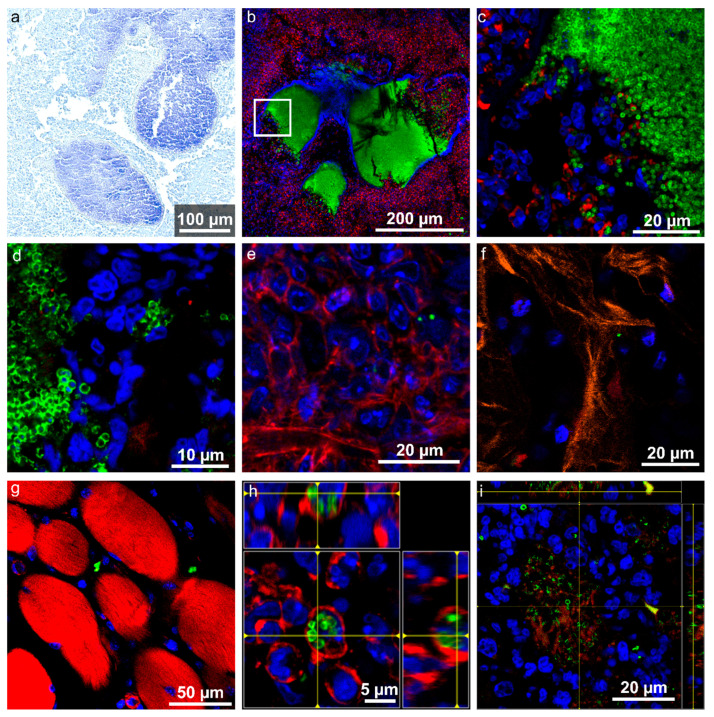
Detailed images of *Staphylococci* found in various host environments. (**a**) Gram-staining of an abscess in the right pelvis (RG1). (**b**) Enlarged view of the SACs in section LF3 shows a dense accumulation of bacteria surrounded by a capsule. The capsule is broken to one side allowing bacteria to spread into the surrounding lesion. The white square is shown in (**c**). (**c**) Enlarged view of bacteria spreading imaged using 2 photon excitation. (**d**) Clusters of bacteria between host cells, originating from RF1 (**e**) Individual *S. aureus* between host cells in inflamed tissue (LF1). (**f**) Occasionally, *S. aureus* were detected in trabecular bone regions (RF1). (**g**) Occasionally, bacteria were also detected in muscle tissue (LF3). (**h**) Intracellular location of *S. aureus* within an immune cell (maybe a neutrophil) as visible from the ortho-view (LF3). (**i**) Bacterial accumulation with actin in the background (RF1). (**b**–**i**) Channel colours for the immunofluorescence images mark the following features (and used fluorophore): green: *S. aureus* (Dy405 or AF488), blue: cell nuclei (DAPI or SYTOXgreen), red: actin-cytoskeleton (I555-Phalloidin), and orange: collagen (SHG).

In the left pelvis, the most prominent bacterial accumulation was found in slice LF3, where three condensed staphylococcal abscess communities (SACs) were already detected in the overview image (Figure 2). Although no deformation was observed on the right pelvis with X-ray imaging (Figure 1), we also detected several SACs in the tissue section of the right pelvis using Gram staining and immunofluorescence imaging (Figure 4a and Supplementary Figures S1 and S6a–d). A dense accumulation of bacteria, which were forming a tightly associated lawn, marks the SACs. Bacteria, but no immune cells, were observed in these central accumulations of staphylococci (Figure 4b). With all imaging contrast modalities (i.e., with H&E (Supplementary Figures S1, S4 and S6a), Gram staining (Figure 4a and Supplementary Figures S1, S6b and S7), and fluorescence imaging (Figure 4b and Supplementary Figure S6c–f)), a capsule was found to surround the SACs. This agrees with published characteristics of SACs where an amorphous pseudocapsule composed of fibrin deposits is found to surround the SACs [36]. In our work, the capsule showed a blue (auto)fluorescence when excited with 405 nm (detection filter 410–513 nm), (Figure 4b and Supplementary Figure S6e). The specific staining of this capsule with DAPI could be excluded by using 2-photon excitation of DAPI (Supplementary Figure S6f, where only very little blue fluorescence was detectable) as well as with SYTOXGreen as DNA stain. For the latter, the surrounding capsule also showed fluorescence signal rather in the blue channel (DyLight405) than in the green DNA-channel (SYTOXGreen), confirming that the blue fluorescence is mainly autofluorescence of the capsule. For most of the SACs found in the pelvis, the capsules were not fully closed anymore, but rather, they had an opening where host immune cells were found to interact with the bacteria (Figure 4c). Outside of the capsule, the SACs were surrounded by inflammatory/granulation tissue with a high infiltration of immune cells and signs of fibrosis stating signs of chronic inflammation as discussed above. Based on the four-stage classification of *S. aureus* abscess formation by Cheng et al. [37,38], these abscesses can be considered to be already at stage four, at which the mature abscess has already ruptured to release bacteria in the surrounding tissue.

Individual *S. aureus* bacteria were also found outside of the SACs in all present tissue types. Significant numbers of individual bacteria were located not only in the inflammatory tissue in close vicinity to the SAC (Figure 4d), but also in the lesion distant to the SAC (e.g., Figure 4e). Most of the bacteria outside of the SACs were found as single bacteria between adjacent host cells (Figure 4e) and also as clusters of bacteria (Figure 4d). In several occasions, bacteria were also found to reside inside of host cells, mainly inside of professional phagocyte cells, such as neutrophils or macrophages. An example is visualised in the orthogonal view in Figure 4h. Most intracellular bacteria were found in the lesion surrounding the SAC, in close vicinity to the opening of the capsule where the abundance of immune cells was found to be highest. Additionally, *S. aureus* clusters were often observed in areas that lacked immune cells but had a background actin staining (Figure 4i), which might be caused by host cell apoptosis, leaving actin behind. Our findings agree with the concept that a *S. aureus* infection may modify the default program of tissue repair by building complex lesions that ensure the pathogen’s persistence in a hostile environment [37]. Such a process is thought to start with a bacterial invasion followed by the attraction of immune cells that are part of the newly formed distinctive lesion, which is often accompanied by the destruction and replacement of the healthy tissue. SACs can establish at the centre of the lesion where they are protected by the surrounding lesion [37,38].

In our experiments, very few bacteria were found in the intact trabecular bone. If bacteria were found in this region, they were mainly in the trabecular spaces (Figure 4f) and not in the mineralised part of the bone. Thus, in this study, we could not identify bacteria inside of osteoblasts or osteocytes as was reported in previous studies with electron microscopy [20]. Smaller numbers of bacteria were also found in the adjacent muscle tissue (Figure 4g and Supplementary Figure S7, panel 6).

**Figure 5:**
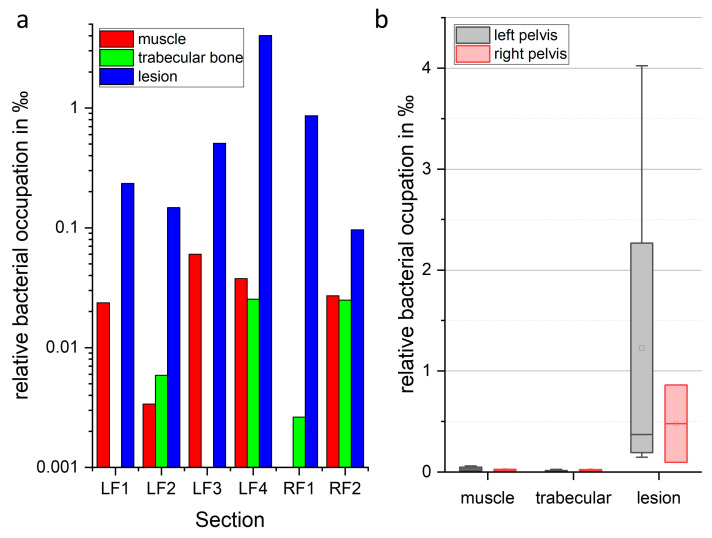
Visualization of bacterial count in the different tissue slices from semi-automated analysis of fluorescence images. (**a**) Relative bacterial occupation (on y axis) of selected tissue types in the individual slices labelled on the x axis. Please note, the y-axis is given in logarithmic scale. Colour codes for tissue region: red: muscle, green: trabecular bone, blue: lesion (when no bar is shown, no bacteria were found in the respective tissue). (**b**) Box plot comparing relative bacterial occupation in the selected tissue types of left and right pelvis from the data of the single slices in (**a**). x-axis: tissue type (grey for left and red for right pelvis), □: mean, central horizontal line: median, upper box line: median 1st quartile, lower box line: median 3rd quartile, error bars: maximal and minimal value. Please note, as there were only 2 slices from right pelvis analysed, there are no error bars on the boxes. (For further information see Supplementary Table S3).

In order to quantify the findings described above, the in-house developed algorithm FindAureus was used to automatically detect and count *S. aureus* in selected regions of the fluorescence images. Supplementary Tables S3 and S4 summarise the results, and Figure 5 presents their illustration as bar plot. It has to be noted that we present a single case study here and values might vary in other cases of chronic osteomyelitis (in particular, as they already vary within one bone in the different sections). As discussed above with the qualitative analysis of the images, most bacteria were found in the lesion where they covered up to 4‰ of the tissue area (slice LF4). Please note that SAC area was excluded from this calculation; otherwise, the relative bacterial occupation in the lesion can reach up to 2.5% in slice F3. The high bacterial load in the lesion agrees with a relatively high bacterial load of *S. aureus* Chwa42 in bone detected by plating bone homogenates of parallel mice of the same experiment in the chronic phase [39]. When comparing the relative bacterial occupation in the lesion of different tissue slices, the particular high value of LF4 falls out while the values obtained for LF1–LF2 and RF1 and RF2 are comparable. Upon a closer investigation of the bacterial distribution in the lesion in the overview images in Supplementary Figure S8, it can be seen that bacteria are not evenly distributed in the lesion, a fact that might be explained by the hematogenous origin of the bone infection. Bacteria are found in higher numbers around oval tissue structures in the lesion. It can be speculated that those regions might be old SACs after rupturing and releasing their bacterial content in the surrounding or otherwise necrotic tissue surrounded by bacteria.

The semi-quantitative assessment also confirmed that lower numbers of bacteria were found in the surrounding muscle tissue as well as in the trabecular bone (Supplementary Table S3, Figure 5).

### 2.6. Biochemical Analysis Using Raman Imaging

**Figure 6:**
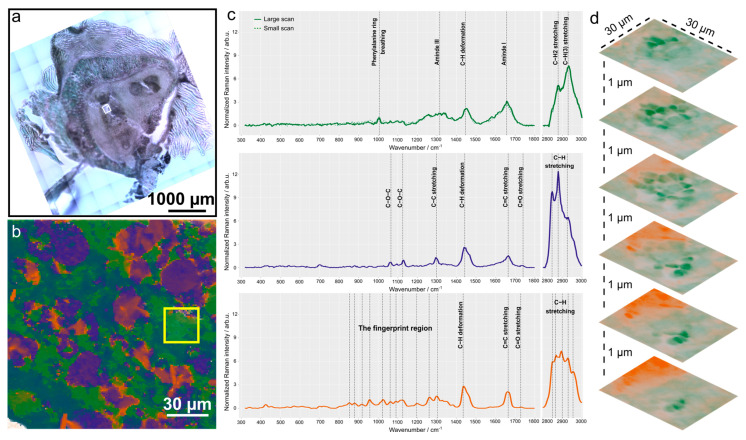
Raman spectroscopic analysis of the lesion. (**a**) Bright field overview image of pelvis section LR1 used for Raman imaging. The small white square indicates the region subjected to Raman imaging. (**b**) False colour Raman image using N-FINDR analysis with intensity-normalised Raman data. (**c**) Endmember (EM) spectra, top row: EM 1, middle row: EM2, and bottom: EM 3. The EM1 of the small scan (dashed line) in also provided in the top row to show the similarities between the two. (**d**) High-resolution 6-layer 3D Raman scan performed in the region highlighted with a yellow square in panel (**b**).

Raman spectroscopic imaging can reveal spatially-resolved fingerprint-like information about the molecular composition of the sample in a label-free and non-destructive manner. For a Raman spectroscopic analysis, we have chosen a tissue section LR1, which is 100 µm apart from section LF3, to be in a tissue region close to the abscess (see Figure 1c). No prior staining or embedding had to be done as with Raman spectroscopy intrinsic information of the sample is probed. The bright field overview image of the investigated section LR1 is depicted in Figure 6a. A region of 150 µm × 150 µm in the lesion was chosen for label-free Raman analysis as in this tissue type most bacteria are expected (see Figure 5). With spectral unmixing using N-FINDR, the false colour Raman image depicted in Figure 6b was generated. Figure 6c highlights the spectral characteristics of the first three endmembers (EM).

The spectral features of EM1 are characteristic for organic material, such as bacteria. At this stage, it is not possible to unambiguously differentiate bacteria from the host tissue. Image scans with a higher spatial resolution are necessary and were recorded (see below). In EM1, protein contributions are visible with the Raman bands around 1655 cm^−1^ (amide I), around 1200–1380 cm^−1^ (amide III), and 1005 cm^−1^ (phenylalanine ring breathing). CH vibrations are visible around 1450 cm^−1^ (C-H deformation vibration) and 2879 cm^−1^ (C-H_2_ stretching of lipids) as well as 2930 cm^−1^ (C-H_(3)_ stretching of proteins). EM1 is scattered through the whole ROI with local accumulations which seem to be composed of smaller, bacteria-sized structures (Supplementary Figure S9).

In order to investigate this further, a high-resolution 3D Raman scan was performed in the region highlighted with a yellow square in Figure 6b. Figure 6d shows the false colour Raman images of this 30 µm × 30 µm large region in 6 different planes; the distance between each plane is 1 µm along the z axis. Here, smaller round features with a diameter of approximately 2–3 µm are also visible when observing endmember 1 in the small scan (Figure 6d). This endmember shows very similar spectral features as EM 1 in Figure 6c, first row. An overlay of both EM with a Raman spectrum of *S. aureus* Chwa 42 recorded from a pure culture [40] is shown in Supplementary Figure S10. When comparing bacteria from a culture and the endmember spectra from inflamed bone tissue, spectral differences are found around 2879 cm^−1^. Raman spectra originating from the bone tissue show a sharp CH_2_ stretching band, which could originate from lipids from the surrounding host cells. Lipid mediators were found to be important to direct immune cells to the infectious focus and be involved in microbial killing [41]. Furthermore, it can be seen that bacteria taken from an in vitro culture show prominent nucleic acid bands (e.g., 788 cm^−1^ and 1588 cm^−1^), indicating an active growth and metabolism in the exponential growth phase. Those bands are hardly visible in the spectra from the tissue samples and point to a metabolically inactive state of the bacteria in the bone slice. The correlation of relative Raman intensity of nucleic acid bands and metabolic activity has been demonstrated for other staphylococci previously [42]. Thus, the observed low intensity of nucleic acid bands points to metabolically inactive bacteria in the lesion tissue. Such inactive bacterial phenotypes could be small colony variants (SCV) that have been described in earlier works [39].

EM 2 (Figure 6c, second row) shows clear spectral features of lipids, such as the prominent and sharp spectral features in the C-H stretching region (2848 cm^−1^, 2879 cm^−1^, 2926 cm^−1^), C-H deformation band (1442 cm^−1^), C-C stretching vibration of aliphatic chains (1298 cm^−1^), and C=C stretching of unsaturated chains (1664 cm^−1^). The spectral features around 1065 cm^−1^ and 1125 cm^−1^ can be assigned to C-O-C vibrations, and the small hump around 1740 cm^−1^ indicates the presence of ester bonds. The spatial distribution of EM 2 shows larger accumulations (around 20 µm in diameter) as well as smaller droplets (Supplementary Figure S9). The similarities of the overall spectral fingerprint of EM2 with the spectra of cells in bone marrow in the intact trabecular bone section recorded in a different region of the bone (section LR2) can be seen in Supplementary Figure S11. Furthermore, a high agreement of EM2 with published spectra of bone marrow adipocytes can be found [43]. This assignment would also agree with the spatial distribution of EM2 (Figure 6b and Supplementary Figure S9).

EM 3 is mainly found as small droplet features, which are clustered in larger regions that could resemble the size of a cell (e.g., a macrophage (Supplementary Figure S9)). The Raman spectrum of EM3 (Figure 6c, third row) is rich in spectral features from lipids, but clearly distinct from EM 2. It has a rich C-H stretching region with Raman bands around 2850 cm^−1^, 2865 cm^−1^, 2900 cm^−1^, 2930 cm^−1^, and 2955 cm^−1^. Furthermore, C-H deformation bands are found around 1434 cm^−1^, C=C stretching around 1664 cm^−1^ and C=O stretching of ester bonds in glycerol esters around 1728 cm^−1^. The fingerprint region shows many well-defined Raman bands, among others around 853 cm^−1^, 879 cm^−1^, 918 cm^−1^, 957 cm^−1^, 1022 cm^−1^, 1061 cm^−1^, 1091 cm^−1^, 1125 cm^−1^, 1197 cm^−1^, 1260 cm^−1^, and 1302 cm^−1^. Some of the spectral features are very similar to lipid droplets found in ex vivo characterization of macrophages [44].

**Figure 7:**
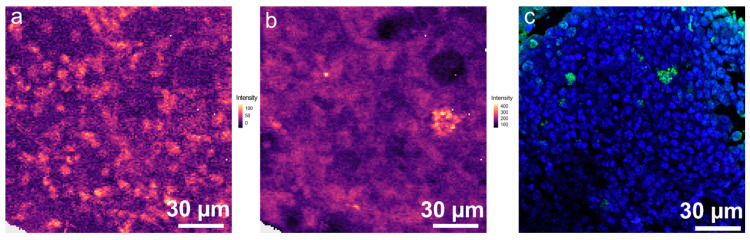
(**a**) False colour Raman image displaying relative intensity of the Raman band at 788 cm^−1^ using non-normalised Raman data. (**b**) False colour Raman image displaying relative intensity of the Raman band at 1580 cm^−1^ using non-normalised Raman data. (**c**) Fluorescence image of the same bone section (but not identical region), stained after Raman analysis. green: *S. aureus* (DY405), blue: nuclei (DAPI), red: actin-cytoskeleton (I555-Phalloidin).

Spectral contributions of nucleic acids were rather low in all endmember spectra. However, they are expected to be present in cell nuclei and also within bacteria. Therefore, false colour Raman images were generated visualizing the relative intensity of the nucleic acid bands at 788 cm^−1^ (Figure 7a) and 1580 cm^−1^ (Figure 7b) using non-normalised Raman data. In Figure 7a bright regions of around 5 µm are visible, which could be the cell nuclei of the host cells. In the heatmap for the Raman band at 1580 cm^−1^ (Figure 7b), similar features are visible as in EM1 (Supplementary Figure S9). With especially high intensity values structures in the size of typical bacteria were visible. After finishing the Raman measurements, bone section LR1 was subjected to fluorescence staining (Supplementary Figure S12 and Figure 7c). It has to be noted that due to technical issues, not the exact very same position is depicted as used for Raman imaging. However, the overall distribution of nuclei and bacterial signal shows excellent agreement with the false colour Raman images (Figure 7). The distribution of the cell nuclei in the fluorescence image resembles the false colour Raman map using nucleic acid distribution in Figure 7a. The bacteria are visualised with specific fluorescent antibodies in Figure 7c and are found throughout the lesion supporting our assignment discussed above.

## 3. Materials and Methods

### 3.1. Hematogenous Osteomyelitis Mouse Model

A previously published mouse model of hematogenous osteomyelitis [45,46] was used in this study. The mouse described in this manuscript was part of a larger study with a total of 11 mice [39]. The animals were maintained according to institutional guidelines in individually ventilated cages and were given food and water ad libitum. General conditional check and weight measurement of the mouse model were supervised by a professional throughout the experiment (6 weeks). This particular mouse was chosen for our study as it presented with a deformed pelvis 6 weeks after infection in the in vivo X-ray images.

Briefly, 1 × 10^6^ CFU (from the log phase of growth) of *S. aureus* strain Chwa42 diluted in 200 µL phosphate-buffered saline (PBS) was injected into the lateral tail vein of a ten-week-old C57BL/6 mouse. The animal was sacrificed by CO_2_ asphyxiation 6 weeks post infection (chronic phase of infection). *S. aureus* strain Chwa42 is a clinical isolate collected from a patient who suffered hematogenous osteomyelitis [39]. *S. aureus* Chwa42 has previously been characterised by means of Raman spectroscopy and its Raman spectrum in comparison to 46 other clinical *S. aureus* isolates that can be found in the supplement of Ebert et al. [40]. Purely by eye, no differentiation of the different isolates is possible as they represent very similar Raman spectra.

All experiments were conducted in accordance with the National Institute of Health Guidelines for the Care and Use of Laboratory Animals (8th edition) and the European Community Council Directive for the Care and Use of Laboratory Animals of 22 September 2010 (2010/63/EU). The study protocol was approved by the competent State Office of Food Safety and Consumer Protection (TLLV, Bad Langensalza, Germany; local registration number: 22-2684-04-02-006/15).

### 3.2. Two-Dimensional X-ray Image Acquisition

The course of infection was monitored weekly from the acute (1 week after infection) to the chronic phase (6 weeks of infection) with X-ray imaging. Whole body X-ray radiography was performed on an In-Vivo Xtreme 16 MP imaging system (Bruker, Ettlingen, Germany) with an exposure time of 3 s. During imaging, animals were anesthetised with 2% isoflurane.

### 3.3. Bone Tissue Preparation and Sectioning

The mouse was sacrificed 6 weeks after induced infection in the chronic phase by CO_2_ asphyxiation. Left and right pelvis were carefully separated from the rest of the bone sections and immersed in 4% paraformaldehyde (PFA) (Sigma-Aldrich/Merck, Taufkirchen, Germany) for 24 h at 4 °C.

The ilium of the left pelvis was further decalcified using 14% ethylenediamine tetraacetic acid (EDTA) (Applichem, Darmstadt, Germany) and 0.2% PFA for 49 days at 4 °C in between changing the solution every 3 to 4 days. Around 140 transverse cryosections from the ilium of 100 µm in thickness were cut using a CM3050S cryostat. The slices were stored at 4 °C in 96 well plates in PBS for further experimentation.

The ilium of the right pelvis was decalcified using decalcifier soft (Carl Roth, Karlsruhe, Germany), which contains 25% EDTA for 24 h, changing the solution after 7 h at room temperature. Subsequently, the tissue was embedded in paraffin and 5 µm sections were cut at the level of infection (Leica RM1265, Nussloch, Germany) for histological staining and immunofluorescence labelling. For comparison, the pelvic bones of an uninfected control mouse were isolated and treated in the same way.

An overview of the tissue slices described in this manuscript is presented in Supplementary Table S1.

### 3.4. Histological Staining

Formalin-fixed paraffin embedded (FFPE) bone slices of the right pelvis of the infected mouse and of the non-infected control mouse were de-paraffinised using xylene (Carl Roth) and hydrated in a series of graded alcohols. Briefly, slides were placed 2× in xylene, 1× in xylene:ethanol (1:1), 2× in 100% ethanol, and subsequently in 95% ethanol, 70% ethanol, and 50% ethanol followed by rinsing with tap water. The slides were left in each solution for 3 min before proceeding to the fresh next solution. Subsequently, bone slices were either stained with hematoxylin and eosin (Mayer’s hemalum solution and Eosin G 0.1%, both Merck, Darmstadt, Germany) to reveal the tissue structure, or Gram staining was performed according to the manufacture’s protocol (Carl Roth, Karlsruhe, Germany) to visualise bacteria.

Bright field images were acquired using an Axio Observer.Z1 (Carl Zeiss, Jena, Germany) equipped with an AxioCam MR R3 camera (Carl Zeiss) and a Plan Apochromat 40×/0.95 objective or a Plan-Neofluar 63×/1.3 oil immersion objective. The whole slices were scanned in tile-scan mode and stitched using the Zen 3.1 blue software (Carl Zeiss).

### 3.5. Immunofluorescence Labelling/Staining

For immunofluorescence labelling, the selected tissue slices were washed once in PBS and blocked for at least 5 h in blocking buffer (2% bovine serum albumin (BSA, Merck), 300 mM glycine (Carl Roth), 2% Triton-X100 (Carl Roth), 20% dimethyl sulfoxide (DMSO, Sigma-Aldrich/Merck), 0.02% sodium azide (Carl Roth)) in PBS containing additionally 5% human serum (Thermo Fisher Scientific, Waltham, MA, USA).

To stain for *S. aureus* and the osteoblast marker osteocalcin, the cryo-sections (100 µm) were first labelled with the primary antibody, rabbit anti-*S. aureus* (rabbit anti–*S. aureus* polyclonal IgG, generated by Squarix Biotechnology, Marl, Germany [47]) in 1:200 dilution plus goat anti-mouse osteocalcin (BioRad, Hercules, California, US) 1:200 dilution in blocking buffer for 10 days at room temperature while shaking. The slices were washed thrice for 1 h and twice for 10 min in permeabilization buffer (20% DMSO, 2% Triton X-100, 0.02% sodium azide in PBS). Next, the secondary antibody, AF488 donkey anti-rabbit Fab fragment (abnova, Taipei, Taiwan) in 1:200 dilution or DyLight405 donkey anti-rabbit IgG (dianova/BIOZOL, Hamburg, Germany) at 1:200 dilution plus Dylight650 donkey anti-goat IgG (Thermo Fisher Scientific) dilution 1:250 in permeabilization buffer were applied for 10 days at room temperature while shaking followed by washing thrice for 1 h and twice for 10 min in permeabilization buffer. Subsequently, I555 phalloidin (abnova) was used at a concentration of 2 rxn/100 µL to label the cytoskeleton and 1 µg/mL DAPI (AppliChem) or 0.5 µM SYTOXGreen (Thermo Fisher Scientific) to visualise cell nuclei. Staining was done for 1 day in blocking buffer while shaking. The slices were washed thrice for 30 min in permeabilization buffer and once for 10 min in distilled water. The samples were carefully placed on glass slides and embedded in VectaShield (Vector Laboratories, Newark, CA, USA) for microscopy.

The FFPE tissue sections (5 µm) from right pelvis were de-paraffinised using xylene and hydrated in a series of graded alcohols as described in Section 3.4. Afterwards, they were washed once in PBS and blocked for 3 h in blocking buffer. Staining was as described above for the cryo-sections, with the only difference that no osteocalcin was stained and staining times were reduced due to the reduced slice size (100 µm vs. 5 µm): for *S. aureus* and respective secondary antibody, staining times were reduced from 10 days to overnight, for phalloidin and SYTOXGreen from 1 day to 5 h.

### 3.6. Confocal Laser Scanning Microscopy (CLSM), Two-Photon Laser Scanning Microscopy and Second Harmonic Generation (SHG) Microscopy

Confocal fluorescence images were acquired using a multiphoton confocal laser scanning microscope (LSM 780 META, Carl Zeiss, Jena, Germany) with Zen 2.2 SP1FP3 (black) software (Carl Zeiss) or a confocal laser scanning microscope (LSM 980 with AiryScan, Carl Zeiss) with Zen 3.6.095.06000 (blue) software. Overview images of the whole slides were generated using the “tile scan” option with a Plan Apochromat 20×/0.8 NA objective. Detailed scans in selected regions were performed using a 63×/1.15 NA water immersion objective (Carl Zeiss) or a 63×/1.4 NA oil immersion objective (Carl Zeiss).

For inspection of bacterial intracellular residency, multiple consecutive images were taken in the axial z-axis of slices (distance between z-planes were 0.5–2 µm). DAPI and DyLight405 were excited with 405 nm (detection range about 410–520 nm); AF488 and SYTOXGreen with 488 nm (detection range about 490–600 nm), IF555 phalloidin with 561 nm (detection range about 565–670 nm), and DyLight650 with 633 nm or 639 nm (detection range about 640–750 nm) using single-photon excitation. To avoid channel crosstalk, only DAPI/DyLight405 and DyLight650 were excited simultaneously; the other channels were recorded as separate tracks. In some detailed scans, collagen was additionally visualised by second harmonics generation (SHG) microscopy using a pulsed Ti:Sa laser (Chameleon, Coherent, Santa Clara, CA, USA) set to 880 nm and detecting backward SHG emission at 437–446 nm [48]. For deeper tissue penetration, some scans were also performed using two-photon excitation microscopy with a pulsed Ti:Sa laser (Chameleon, Coherent) set to 775 nm or 790 nm as the excitation source and collecting the emission with the same channel settings but through maximal pinhole diameter or collecting DAPI/DyLight 405 emission using a 485 nm SP filter, AF488/SYTOXGreen emission with a 525/50 BP filter and AF555 emission with a 580/40 BP filter with non-descanned detection (NDD).

### 3.7. Fluorescence Images Analysis

For manual image viewing and analysis, Fiji (ImageJ v1.53t, [49]) and Zen 3.4 (blue edition, Carl Zeiss) were used. Stitching of individual tiles was performed using Zen 3.4 (blue edition, Carl Zeiss) or the Fiji Stitching Plugin [50]. Automated detection and counting of the bacterial regions across a voxel were employed using a novel in-house algorithm scripted in Python language [51].

### 3.8. Raman Spectroscopic Imaging

For Raman spectroscopic characterization tissue slices were transferred onto alginate-coated (with 0.75% alginate solution using alginic acid sodium salt, from brown algae, Sigma-Aldrich/Merck, Taufkirchen, Germany) CaF_2_ slides (Crystal, Berlin, Germany) and immersed in 0.5 M CaCl_2_ solution (Carl Roth, Karlsruhe, Germany) in a petri dish.

An upright confocal Raman microscope (alpha300R, WITec, Ulm, Germany) equipped with a frequency-doubled Nd YAG solid-state laser (532 nm) was used. Laser power was adjusted to 15 mW in the sample plane. The scattered light was collected through the illumination objective (60× water immersion objective, NA, 1.0, Nikon; Tokio, Japan) and guided with an optical fiber of 100 µm diameter to the spectrometer. A 600 g/mm grating served to diffract the Raman scattered light prior to recording on a CCD camera (spectral centre set to 2200 cm^−1^, resulting in a spectral window of 50–3888 cm^−1^). Raman spectra of silicon wafer and 4-acetamidophenol were recorded prior to the measurement for calibration purposes.

Bright-field images of the whole slices (5000 µm × 5000 µm) were acquired for locating/relocating the regions of interest (ROI) with a 10× objective (NA, 0.3, Nikon). For in-depth spectral characterization, a ROI within the lesion from slice LR1 and of an intact trabecular region from slice LR2 were chosen. In the lesion, an overview Raman spectroscopic image was recorded, covering 150 µm × 150 µm with a step size of 1 µm and 2 s integration time per spectrum. Within this ROI, a high-resolution Raman image of 30 µm × 30 µm was recorded with a reduced step size of 0.25 µm. In addition, spectral acquisition covered six z-planes with a distance of 1 µm along the z-axis between each image. To characterise trabecular bone tissue, a section of 100 µm × 100 µm was chosen in slice LR2 and imaged in 3 layers spanning 6 µm in depth with a step size of 1 µm in xy-direction and an integration time of 2 s per point.

### 3.9. Analysis of the Raman Image Scans

Processing and analysis of the Raman image scans were done using R (version 4.1), R Studio (version 1.4) IDE and the following packages: ggplot2 [52] for visualisations, hyperSpec [53] and dplyr [54] for data import and organization, matrixStats [55] for optimised matrix operations, unmixR [56] for N-FINDR algorithm.

In total, three Raman image scans were analysed: the ROI covering 150 µm × 150 µm in LR1, the high-resolution Raman image within that ROI (i.e., 30 µm × 30 µm with 6 z-planes), and intact trabecular bone section in LR2 (100 µm × 100 µm). First, all spectra were pre-processed in the following way: cosmic ray noise was automatically corrected by algorithm described in Ryabchykov, O. et al. [57], baseline was corrected by applying SNIP [58] algorithm using 35 iterations, and the silent region 1800–2800 cm^−1^ was cut out.

Linear spectral unmixing was performed using N-FINDR algorithm [59]. By assuming that each spectrum (i.e., a pixel in a Raman image scan) is a linear mixture of some pure components unknown beforehand, spectral unmixing allows for extraction of those pure components (also called endmembers) with following decomposition of the image by their abundances. N-FINDR algorithm is an endmember extraction approach based on the assumption that there is at least one pure pixel for each endmember. The original work [59] also demonstrates that this assumption can be violated to some degree. Once the endmembers were found, their relative abundances in each pixel can be calculated by non-negative least squares (NNLS). The resulting false colour Raman image reflects the calculated abundances.

Since the number of endmembers in the image is unknown beforehand, it was estimated by running the algorithm multiple times with different number of endmembers and the best output was chosen. With this approach it was found that three or four endmembers were sufficient for our data set.

### 3.10. Statistical Analyis

Quantification of bacterial occupation was performed from the overview fluorescence images of all investigated slices (LF1-LF4 and RF1-RF2). Therefore, the different tissue types (lesion, trabecular bone, muscle, vascularised tissue, perichondrium, bone marrow) were predefined and marked manually using visual inspection. The area occupied by bacteria was determined from the fluorescence channel representing the *S. aureus*-specific antibody and related to the total tissue area per slice. For comparison of left and right pelvis, the area of the different tissue types as well as the fraction of area occupied by bacteria were averaged for the left and for the right pelvis from the investigated sections. All data are given as mean ± standard deviation (S.D.). For box plots (generated with OriginPro2019), additionally the median and the 1st and 3rd quartile were calculated. The 1st quartile value is given as the median of the upper half of the data while the 3rd quartile value is given as the median of the lower half of the data.

## 4. Conclusions

In this study, we employed a hematogenous osteomyelitis mouse model to investigate this condition on different imaging length scales. Alterations in the behaviour and movement patterns of the mouse mirrored the complications seen in humans with osteomyelitis. An in-depth analysis explained the cause of the movement restrictions with a deformed bone architecture seen in an X-ray analysis and extensive lesions observed on a microscopic level.

We employed advanced imaging techniques to characterise a large region of the ilium of a mouse with pelvis osteomyelitis. Significant osseous changes were detected at a microscopic scale. In the highly deformed left part of the pelvis, staphylococcal abscess communities (SACs) and lesion tissue could be detected and dominated the bone. Similar bone tissue changes had been previously reported for chronically florid osteomyelitis. It was further found that the infection was not homogeneously distributed in the bone and also that slices with almost intact healthy bone tissue with a high osteoblastic activity at the outer trabecular bone regions were found in between slices with severe osteomyelitis-induced osseous changes.

A calculation of the bone area in the tissue slices reflected that the left pelvis was larger than the right pelvis (factor of ~1.6). This is in agreement with X-ray observations. Interestingly, on a cellular level, the right pelvis was also highly affected by the *S. aureus* infection and abscesses, and large regions of lesions were found here as well. This indicates that osteomyelitis-induced osseous changes and cellular remodelling starts well before changes are detectable by non-invasive whole body imaging, such as an X-ray, and points to one of the large difficulties also reported for fast diagnosis in humans. Thus, tissue biopsies for bone histology are still reported in the literature as the diagnostic standard for osteomyelitis.

Specifically, fluorescence imaging techniques were able to detect the presence of staphylococcal abscess communities (SACs), as well as a single bacteria and small bacterial accumulations in the lesion within the bone tissue. Smaller numbers of bacteria were also found in adjacent muscle tissue. This information is crucial for understanding the spread and impact of bacteria in the affected area. Furthermore, our use of label-free Raman spectroscopic imaging allowed us to reveal the presence of bacteria in bone slices. Through the spectral unmixing technique, we were able to characterise these bacteria as metabolically inactive, representing the same metabolic status as reported for metabolically silent small colony variants (SCVs).

Together, our advanced imaging techniques have provided a comprehensive understanding of the bacterial behaviour and tissue changes associated with bone infections.

## Data Availability

Data is available upon request.

## Supplementary Materials

The supporting information can be downloaded at: https://www.mdpi.com/article/10.3390/ijms24119762/s1.
