# Supplementary material for: Insights into S. aureus-Induced Bone Deformation in a Mouse Model of Chronic Osteomyelitis Using Fluorescence and Raman Imaging
Source: Int J Mol Sci. 2023 Jun 5;24(11):9762. doi: 10.3390/ijms24119762 (PMC10254067; doi:10.3390/ijms24119762)
Supplement: Supplementary file 1 [file ijms-24-09762-s001.zip › ijms-2422060-supplementary.pdf]

Insights into *S. aureus*-induced bone deformation in a mouse model of chronic osteomyelitis using fluorescence and Raman imaging

Shibarjun Mandal[1], Astrid Tannert[1,2], Christina Ebert[1,2], Rustam R. Guliev[1], Yvonne Ozegowski[2,3], Lina Carvalho[4], Britt Wildemann[5], Simone Eiserloh[1,2], Sina M. Coldewey[2,6], Bettina Löffler[2,3], Luís Bastião Silva[7], Verena Hoerr[3,8], Lorena Tuchscherr[2,3], Ute Neugebauer[1,2,9]

1 Leibniz Institute of Photonic Technology (Member of Leibniz Health Technologies, Member of the Leibniz Centre for Photonics in Infection Research, LPI), 07745 Jena, Germany
2 Center for Sepsis Control and Care, Jena University Hospital, 07747 Jena, Germany; lorena.tuchscherrdehauschopp@med.uni-jena.de (L.T.)
3 Institute for Medical Microbiology, Jena University Hospital, 07747 Jena, Germany
4 Institute of Anatomical and Molecular Pathology, Faculty of Medicine, University of Coimbra, 3004-504 Coimbra, Portugal
5 Experimental Trauma Surgery, Jena University Hospital, 07747 Jena, Germany
6 Department of Anaesthesiology and Intensive Care Medicine, Jena University Hospital, 07747 Jena, Germany
7 BMD Software, PCI-Creative Science Park, 3830-352 Ílhavo, Portugal; bastiao@bmd-software.com
8 Heart Center Bonn, Department of Internal Medicine II, University Hospital Bonn, 53127 Bonn, Germany
9 Institute of Physical Chemistry and Abbe Center of Photonics, Friedrich Schiller University Jena, 07743 Jena, Germany

Content

Supplementary Material and Methods. 3
Experimental details of control bone from a healthy mouse 3
Supplementary Graph 3
Supplementary Graph S1: Weight of the mouse during the course of the infection 3
Supplementary Figures 4
Supplementary Figure S1: Overview images of tissue slices of the right pelvis 4
Supplementary Figure S2: Osteocalcin and collagen deposition at different regions in infected bone 5
Supplementary Figure S3: Osteocalcin deposition observed in selected tissue slices 6
Supplementary Figure S4: Haematoxylin and eosin images of infected and healthy pelvis 7
Supplementary Figure S5: Signs of hyper-vascularized tissue 8
Supplementary Figure S6: Images of SACs 9
Supplementary Figure S7: Gram staining of a paraffin section of the right pelvis 10
Supplementary Figure S8: Overview fluorescence images highlighting bacteria distribution in the lesion 11
Supplementary Figure S9. Distribution of endmember abundances for the large scan Raman image 12
Supplementary Figure S10. Overlay of endmember spectra with pure bacteria spectra 12
Supplementary Figure S11: Raman spectroscopic analysis of the uninfected trabecular bone region 13

Supplementary Figure S12: Overview image of tissue section LR1: 14
Supplemental Tables 15
Supplementary Table S1: Overview of tissue slices described in the manuscript 15
Supplementary Table S2: Approximated area of bone tissue 15
Supplementary Table S3: Approximated area and bacterial region count for muscle tissue, trabecular bone and the lesion with inflamed tissue 16
Supplementary Table S4: Approximated area and bacterial region count in other tissue types found in specific slices 16

Supplementary Material and Methods.

Experimental details of control bone from a healthy mouse

As control served bones obtained from a healthy, untreated mouse (C57BL/6, 82 weeks, male). The mouse was sacrificed by cervical dislocation.

The left pelvis was carefully separated from the rest of the bone sections, and immersed in 4 % paraformaldehyde (PFA) (Sigma-Aldrich/Merck, Taufkirchen, Germany) for 24 hours at 4° C. Decalcification and further handling for histological staining was performed as outlined in section 2.3 and 2.4 in the main manuscript for the right pelvis section of the osteomyelitis mouse.

Supplementary Graph

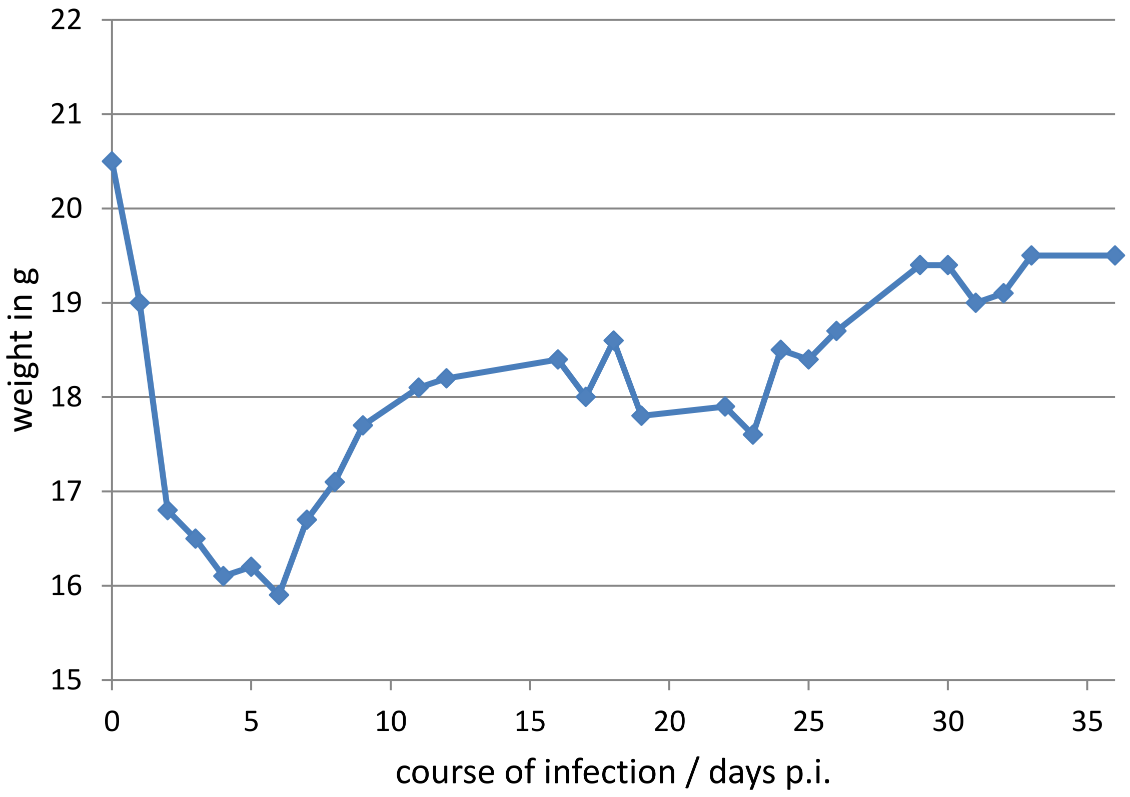

Supplementary Graph S1: Weight of the mouse during the course of the infection. Weight was assessed once per day (except on weekends in the later experimental phase). Day 0 marks the day of infection. The calculated mean weight loss (± standard deviation) during the six days with the lowest weight (day 2 to day 7 p.i.) was 20.2 ± 1.7 %. After the acute phase the mouse regained weight again and reached around 95% of its initial weight (mean weight with standard deviation of the last six values, i. e. day 29 to day 36 p.i., is 94.2±1.0 %).

Supplementary Figures

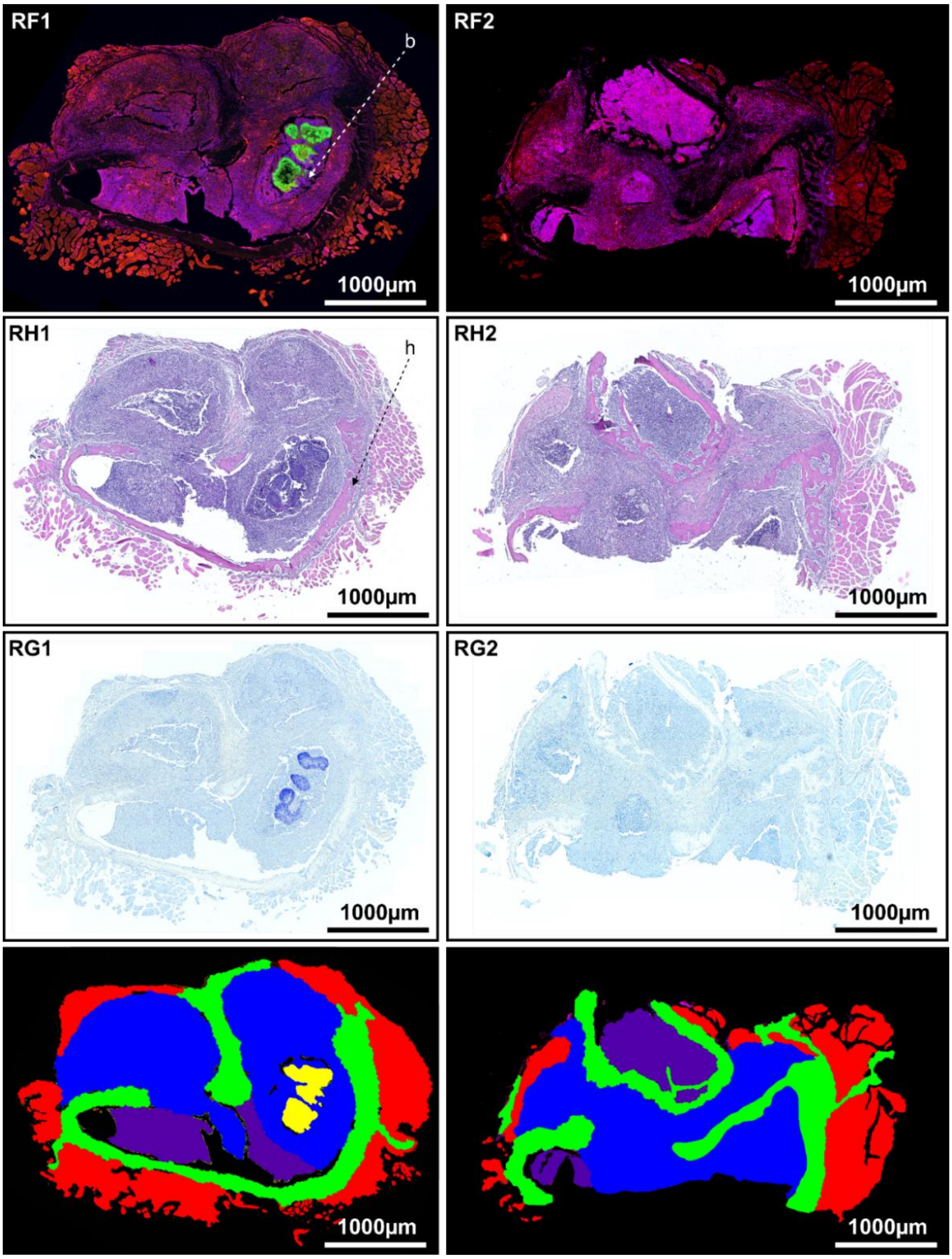

Supplementary Figure S1: Overview images of tissue slices of the right pelvis (paraffin sections) in two different sections R1 (left column) and R2 (right column).

RF1 and RF2: Confocal images of immunofluorescence-labelled sections. Blue: Cell nuclei stained with SytoxGreen, red: actin-cytoskeleton, green: *S. aureus* (specific antibody, secondary antibody Dy405).

RH1 and RH2: Hematoxylin and eosin-stained images.

RG1 and RG1: Gram-stained images.

Last row: Manually colour-coded slices of bone tissue used for calculating the area of the respective tissue type (see Supplementary Table S2) and abundance of bacteria (see Supplementary Table S3), red: muscle tissue, green: trabecular bone, blue: lesion, violet: bone marrow, yellow: Staphylococcal abscess communities (SACs).

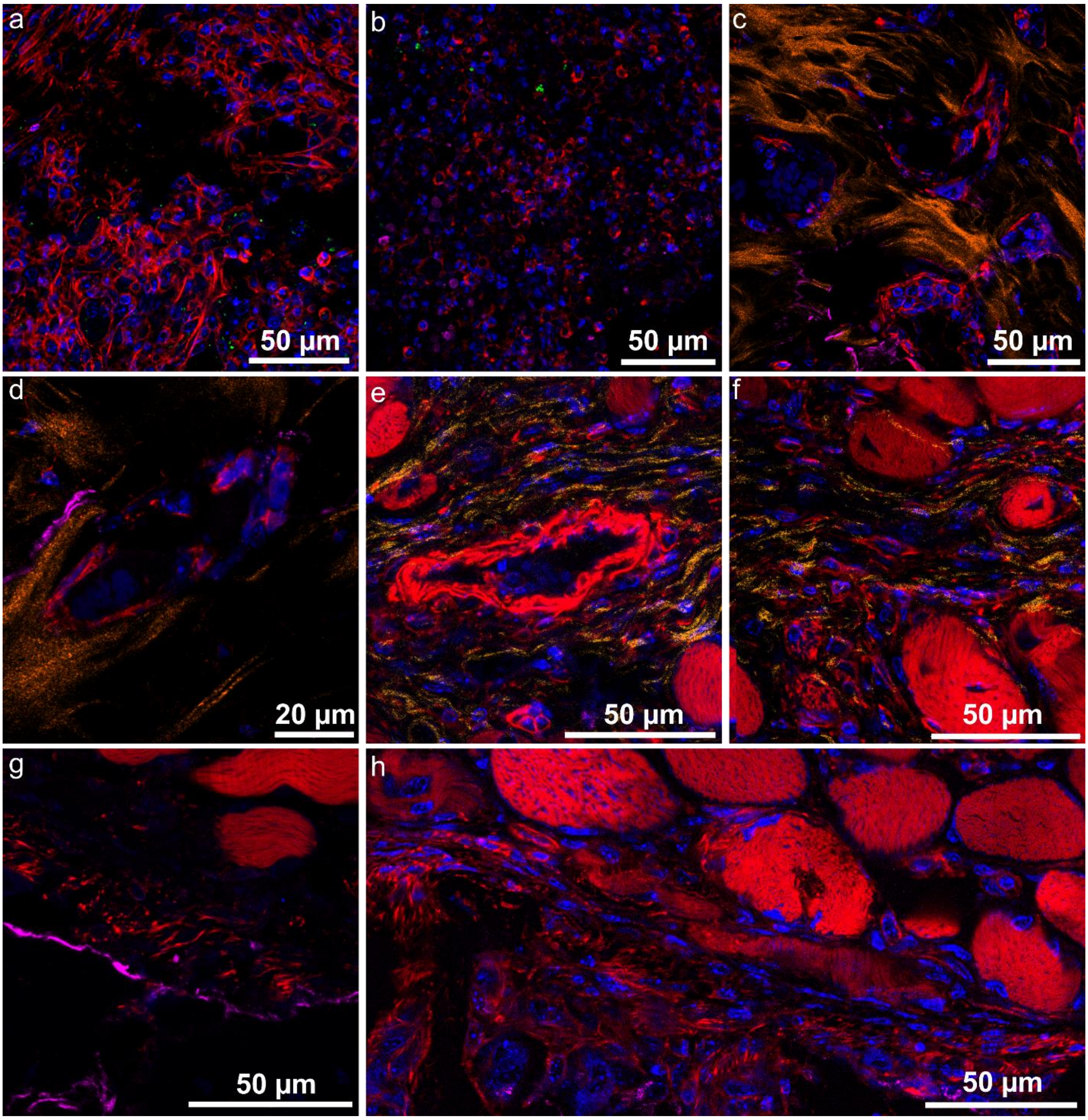

Supplementary Figure S2: Osteocalcin and collagen deposition at different regions in infected bone (immunofluorescence staining): **a) and b)** lesion tissue in LF3, LF4, **c) and d)** trabecular bone of section LF2, LF4, **e)** and **f)** muscle infiltrated with lesion tissue in LF3, **g) and h)** periosteal region in LF4 and LF3.

Colours decode the following channels: blue: DNA/cell nuclei (DAPI), green: *S. aureus (*Alexa 488), red: actin cytoskeleton (I555 phalloidin), pink: osteocalcin (DY650), orange: collagen (SHG microscopy).

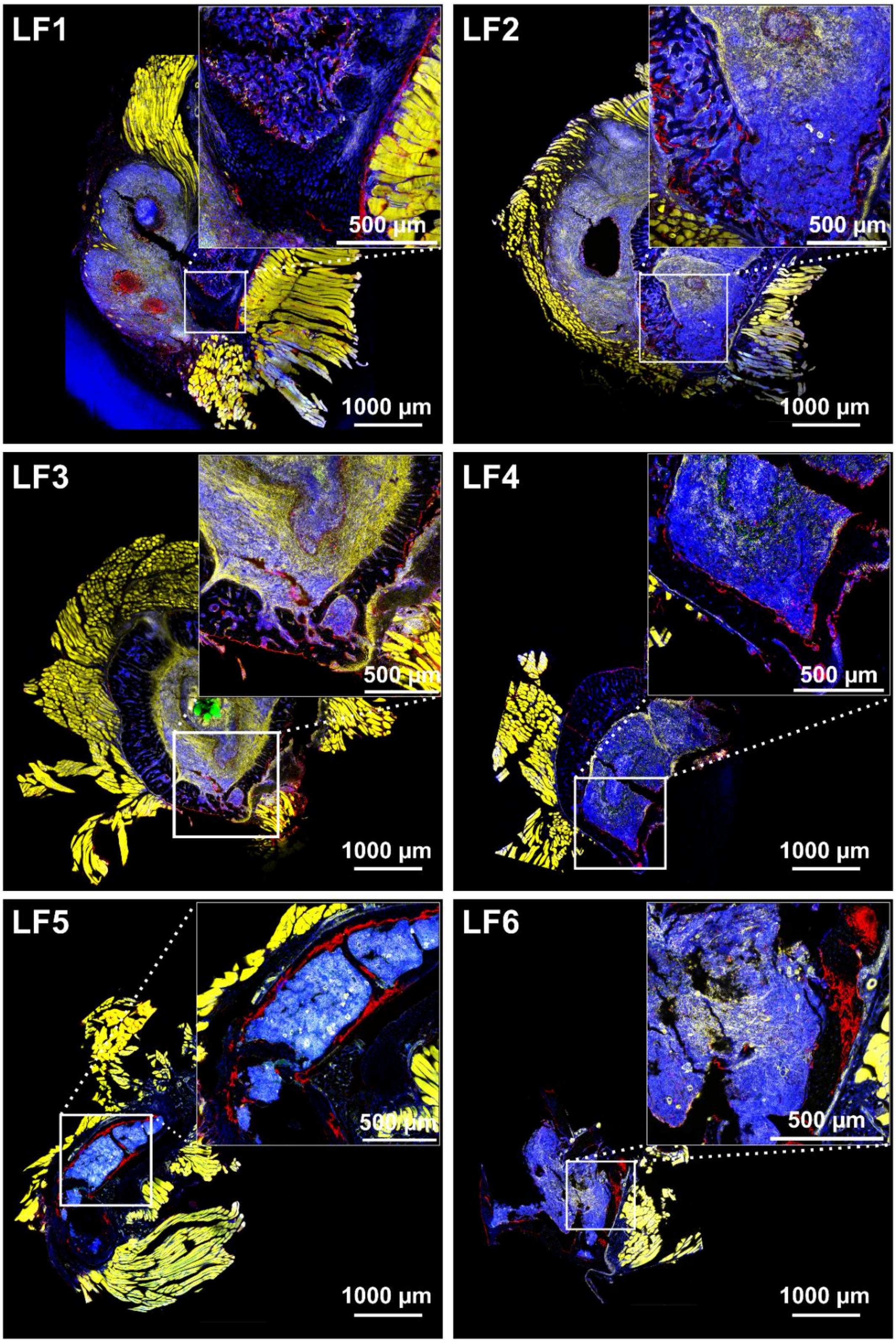

Supplementary Figure S3: Osteocalcin deposition observed in selected tissue slices. The selected tissue slices LF1-LF6 exhibit the presence of osteocalcin, which serves as a marker of osteoblastic activity. The inset provides an enlarged view of the osteocalcin deposition.

Please note, colour code is different than in most fluorescence images shown in the manuscript. Here, osteocalcin is displayed in red (DY650), DNA/cell nuclei are blue (DAPI), the actin cytoskeleton in yellow (I555 phalloidin), and *S. aureus* is coloured green (Alexa 488).

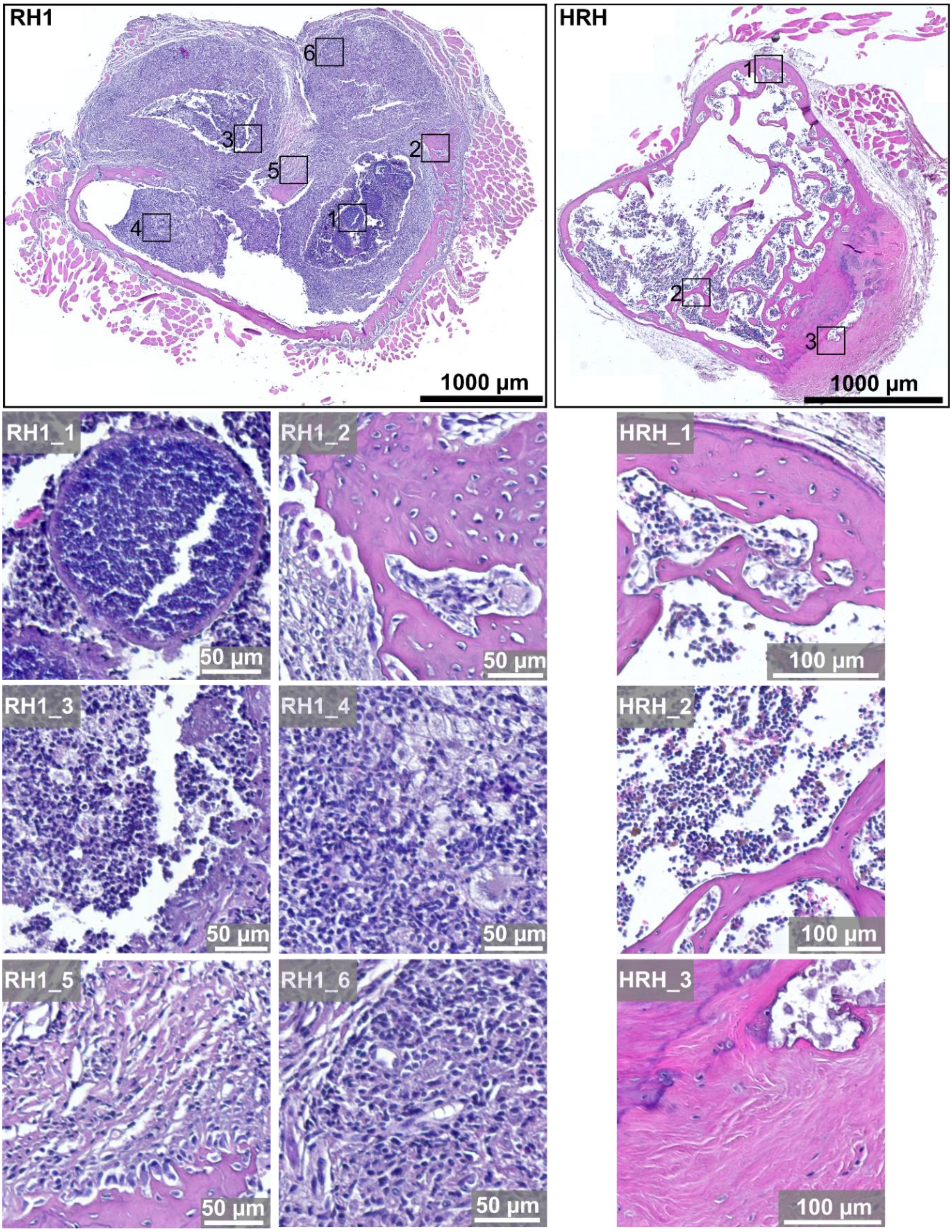

Supplementary Figure S4: Haematoxylin and eosin images of infected and healthy pelvis. ***Left column*** shows H&E images of the right pelvis (RH1) from the mouse shown in Figure 1, main manuscript. Small squares indicate regions shown enlarged below: **RH1_1**) SAC, **RH1_2**) Trabecular space, **RH1_3/4/5/6)** Four different regions within the lesion where nuclei, macrophages, and fibroblasts accumulate. The ***right column*** shows respective images from a healthy control mouse (HRH)**. HRH1_1/2/3)** Regular spongy bone with the presence of regular trabecular spaces and bone marrow cells.

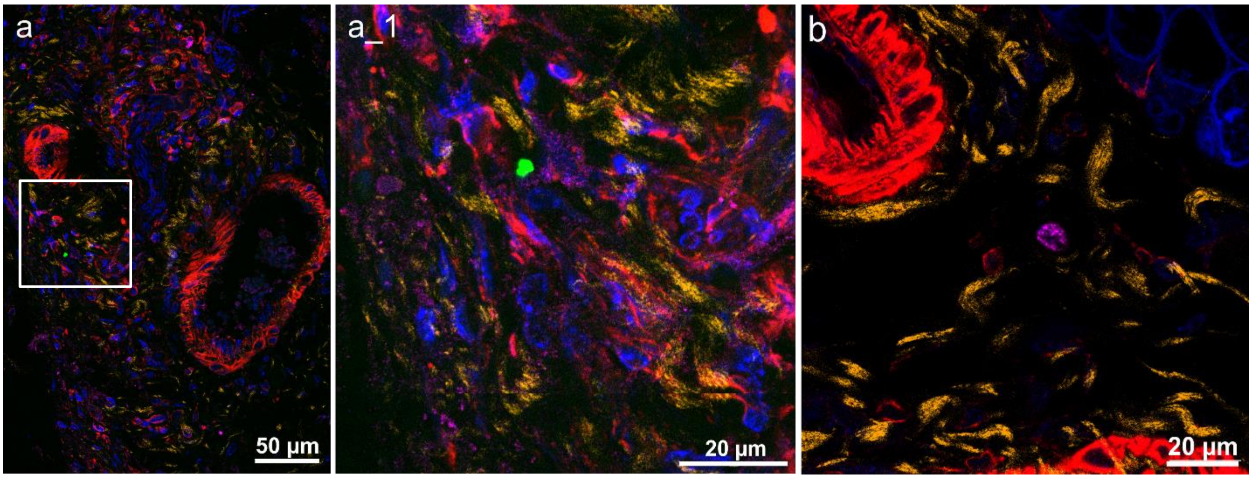

Supplementary Figure S5: Signs of hyper-vascularized tissue were found especially in slice LF1 (cyan region in Figure 2, 2nd Row, from the main manuscript). **a, b)** Representative images demonstrating hyper-vascularized tissue with evident blood vessels. **a_1)** Magnified region from (a) as indicated in panel a) with a white square. This region displays also a cluster of bacteria also present within the tissue. Colours decode the following fluorescence channels: blue: DNA/cell nuclei (DAPI), green: *S. aureus* (Alexa 488), red: actin cytoskeleton (I555 phalloidin), pink: osteocalcin (DY650), orange: collagen (SHG microscopy).

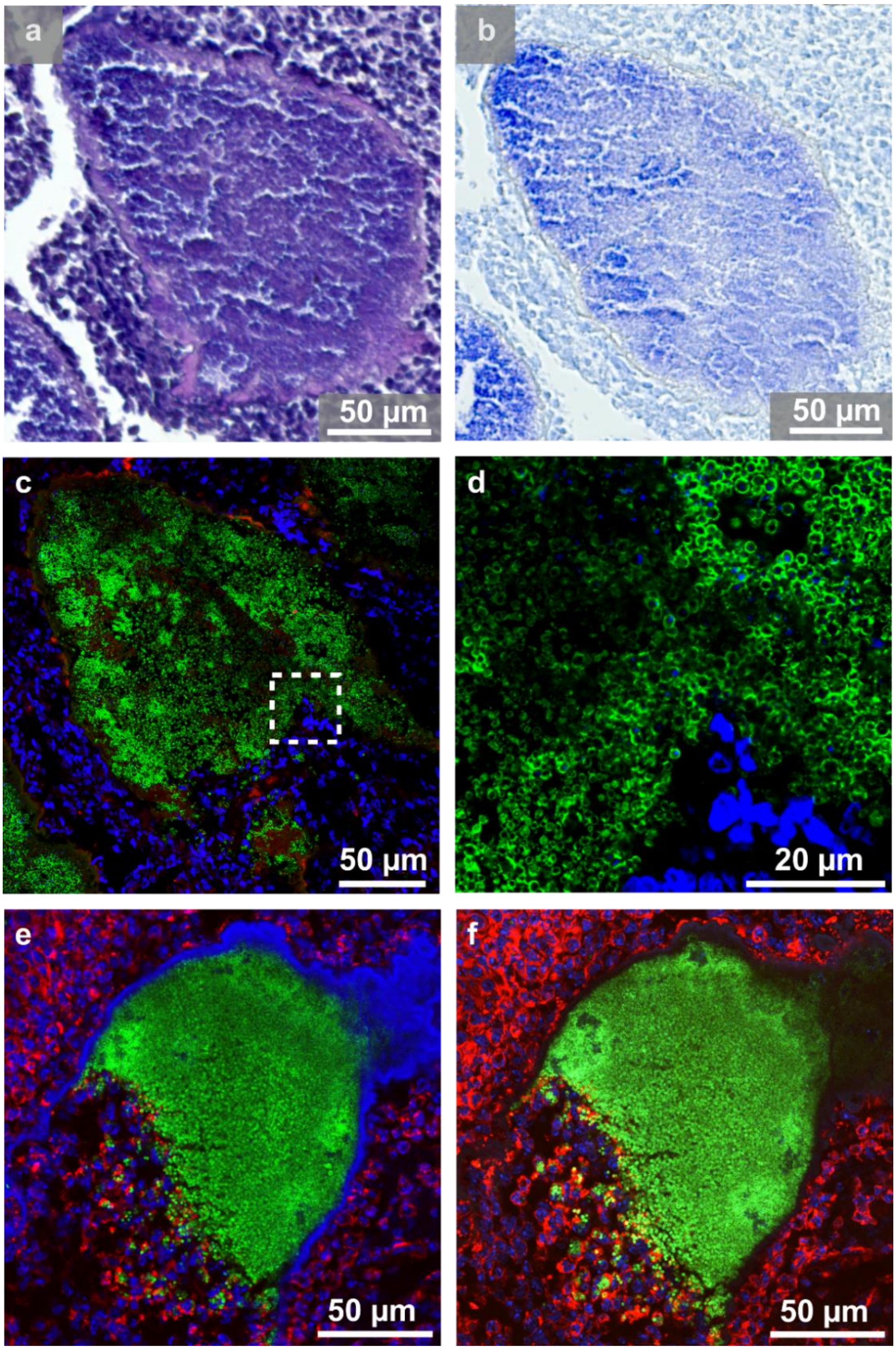

Supplementary Figure S6: Images of SACs. a) H&E staining of an encapsulated abscess found in the right pelvis section (RH1). **b)** Gram-staining of the same abscess in a parallel slice (RG1). **c)** Immunofluorescence of the encapsulated *S. aureus*-filled abscess of the right pelvis (RF1, confocal images, single-photon excitation) with **d)** detailed view of the area in the white square. **e)** Immunofluorescence of an encapsulated abscess in the left pelvis section (LF3, confocal image, single-photon excitation and **f)** same slice as in (e) imaged with two-photon excitation. Colors in the immunofluorescence images decode the following channels: blue: DNA/cell nuclei (SYTOXgreen in (c) and (d), DAPI in (e) and (f)), green: *S. aureus* (DY405 in (c) and (d), Alexa 488 in (e) and (f)), red: actin cytoskeleton (I555 phalloidin).

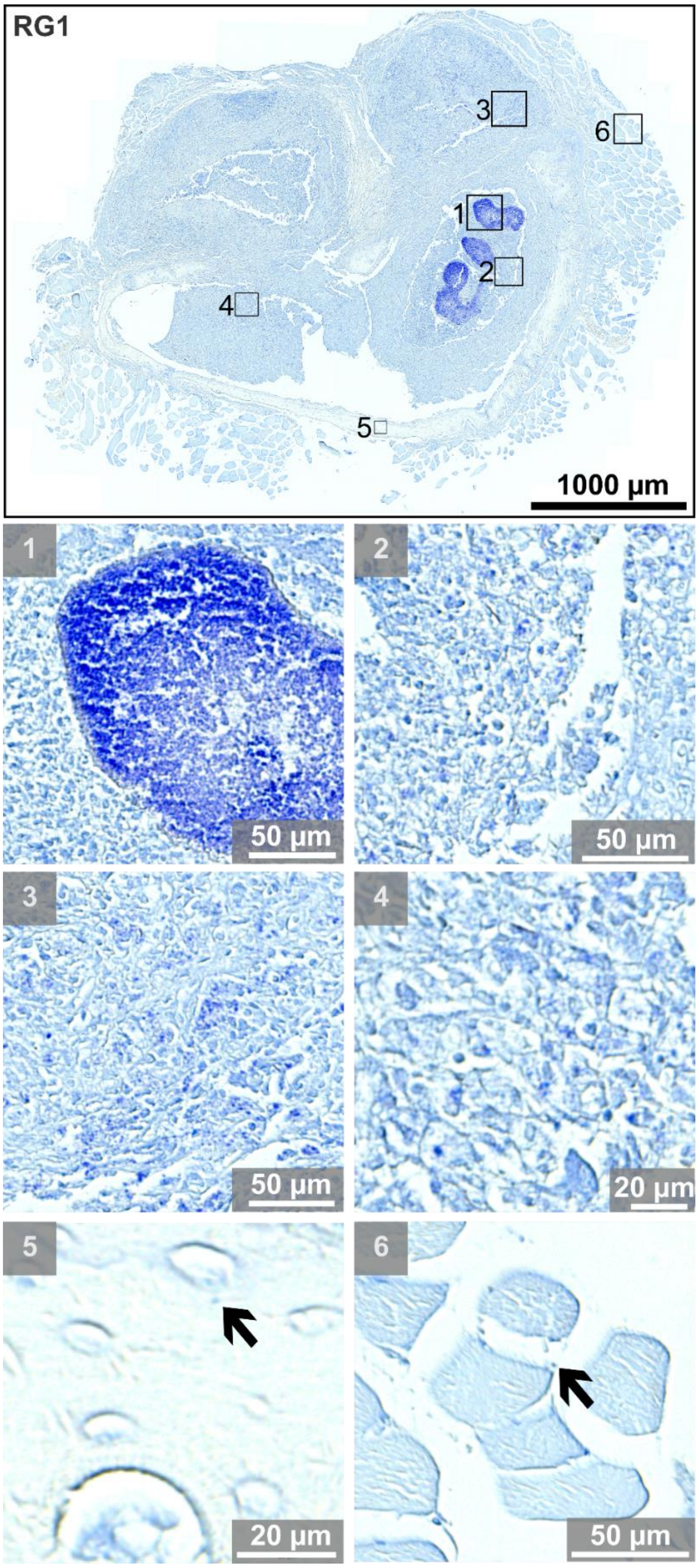

Supplementary Figure S7: Gram staining of a paraffin section of the right pelvis (RG1). The different enlarged panels from the indicated areas highlight the presence of bacteria (blue): **1-4**: bacteria-filled abscess, different regions with many bacteria within the lesion, **5**: single bacterium in trabecular bone (arrow), **6**: single bacterium in muscle tissue (arrow).

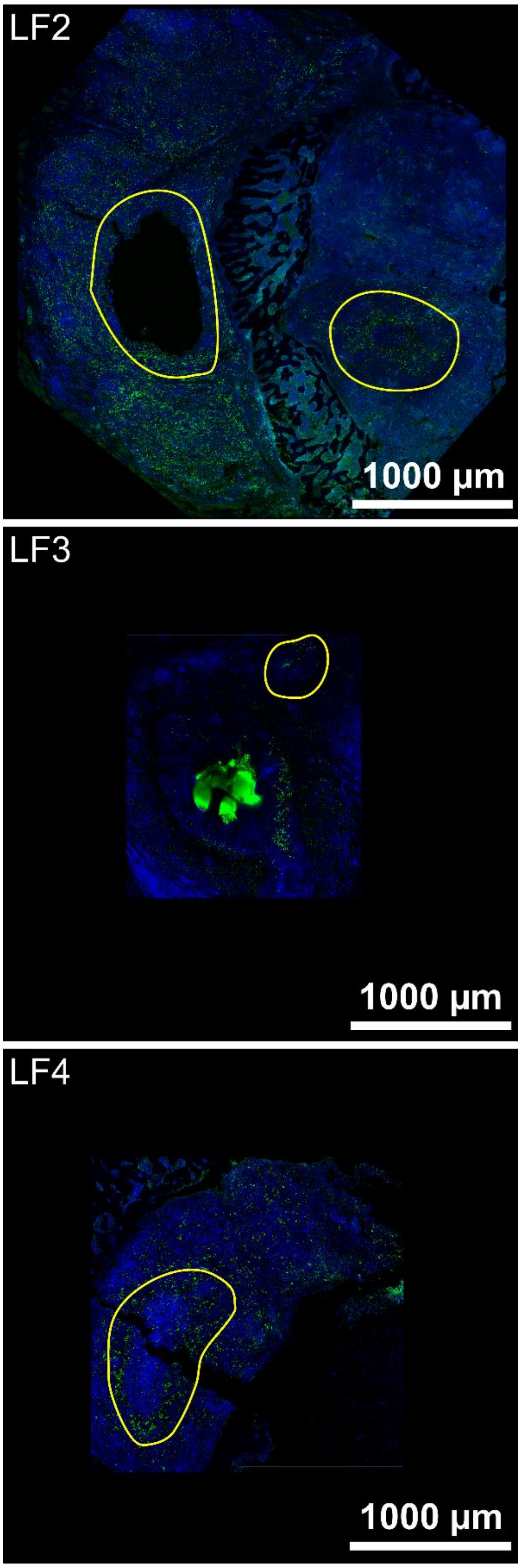

Supplementary Figure S8: Overview fluorescence images highlighting bacteria distribution in the lesion of tissue slices LF2, LF3 and LF4. Bacteria are stained in green, and nucleic acids (e.g. in host cell nuclei) in blue. Most individual bacteria are found around oval tissue irregularities (marked with yellow contours) that might have been previously a SAC or necrotic tissue.

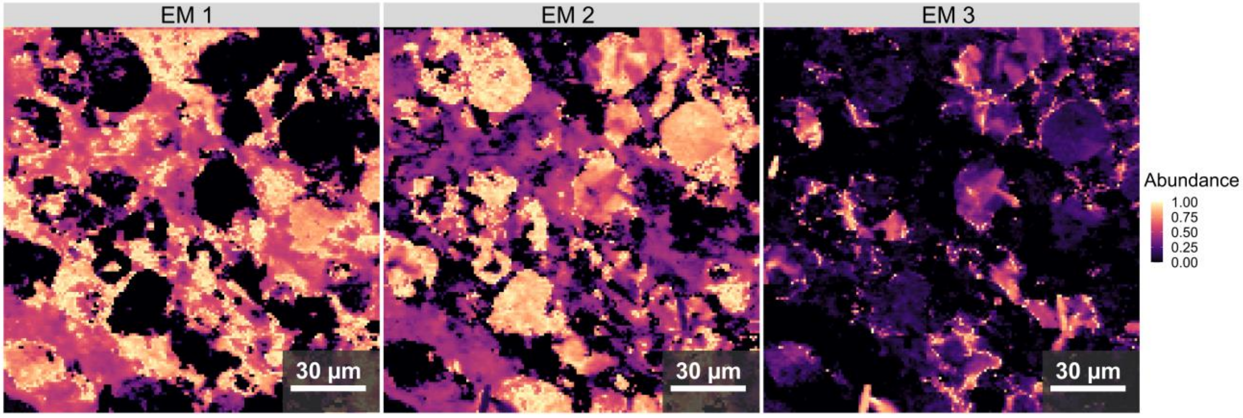

Supplementary Figure S9. Distribution of endmember abundances for the large scan Raman image (the region of 150 µm x 150 µm. The colors are in a gradient from black (corresponds to abundance 0) to white (corresponds to abundance 1).

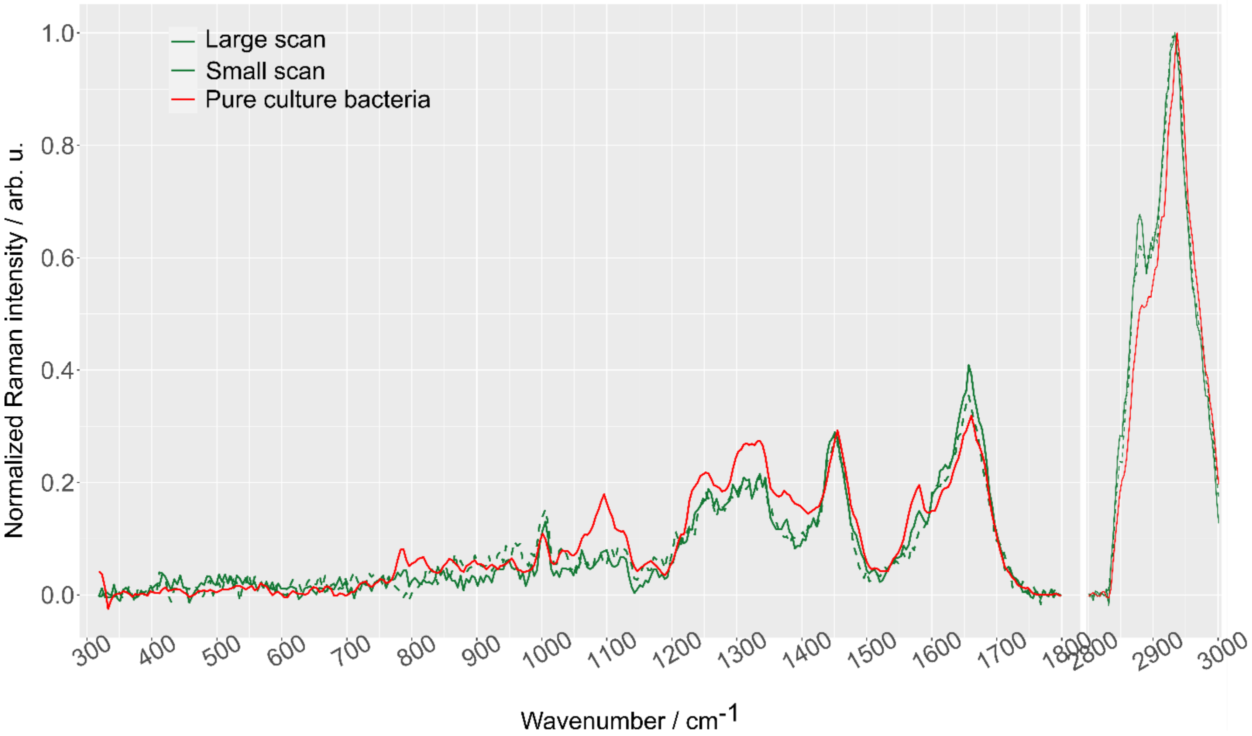

Supplementary Figure S10. Overlay of endmember spectra with pure bacteria spectra: EM1 from the large scan (green solid line), EM1 from the small scan (green dashed line), and the Raman spectrum of *S. aureus* Chwa 42 recorded from pure culture (red solid line). The data for the pure culture spectrum is taken from the previous work [Ebert et al. J. Raman Spectrosc. 52 (2021) 2660-2670].

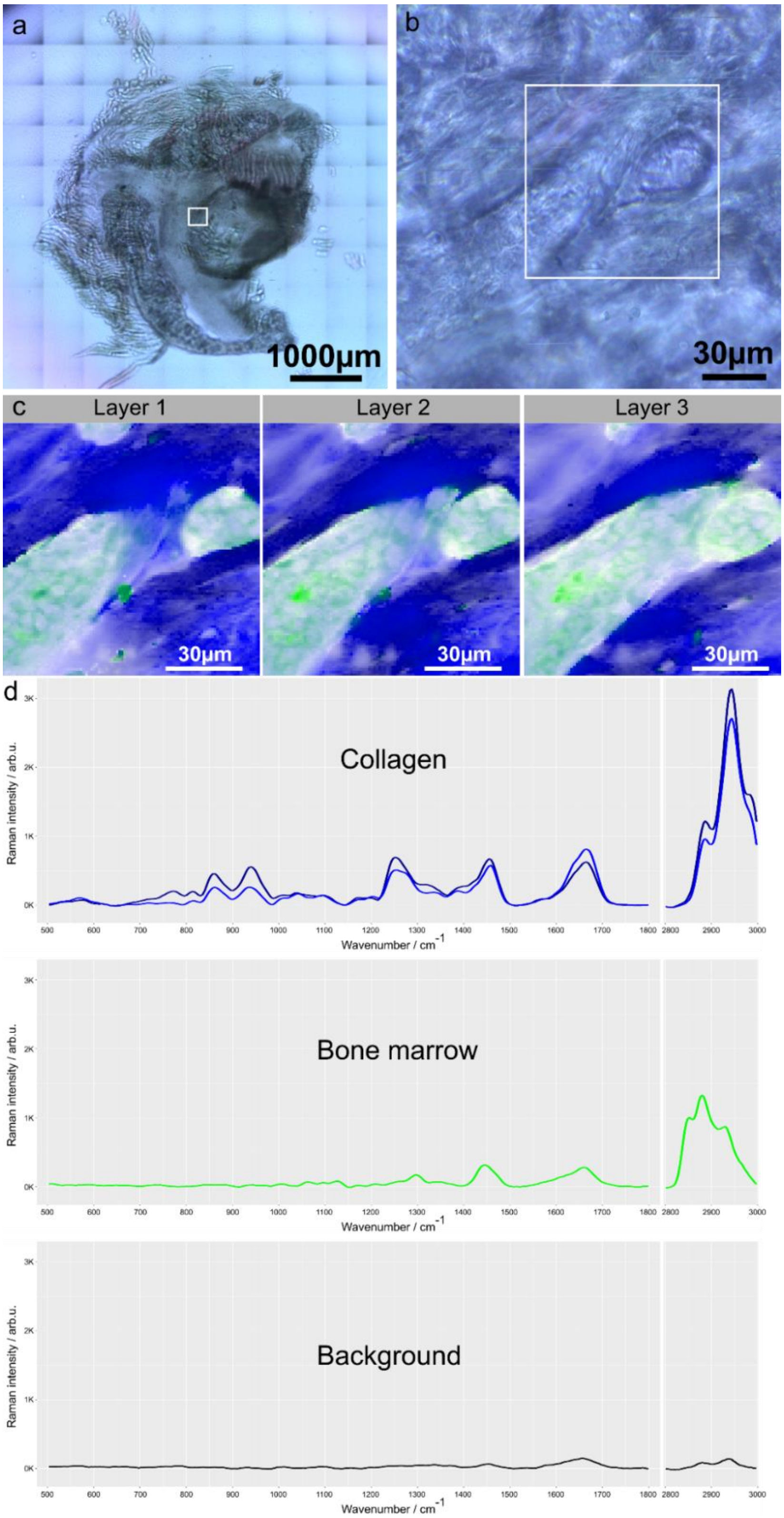

Supplementary Figure S11: Raman spectroscopic analysis of the uninfected trabecular bone region. **a)** Bright field overview image of pelvis section LR2 used for Raman imaging, white square indicates the selected region. **b)** Enlarged view of the selected region, white square indicates the region subjected to Raman imaging. **c)** High-resolution 3-layer Raman scan performed in the region highlighted in panel (b). **d)** Endmember spectra for collagen (top), bone marrow (middle), and background (low).

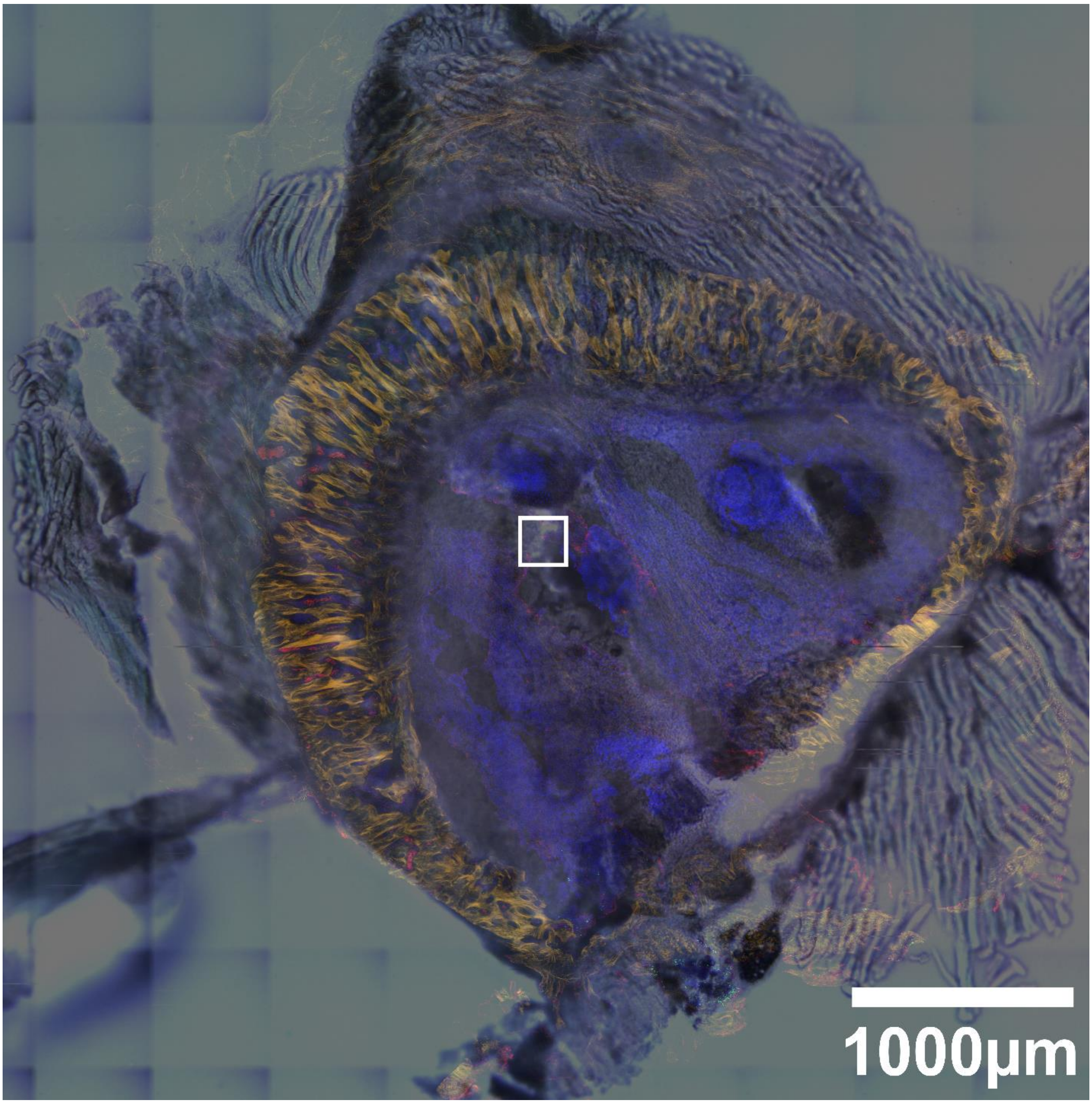

Supplementary Figure S12: Overview image of tissue section LR1: Overlay of bright field and fluorescence image after staining. After Raman analysis, section LR1 was subjected to fluorescence staining: blue: DAPI (cell nuclei), green: DY405 (*S. aureus*), red: I555 phalloidin (actin cytoskeleton) orange: SHG microscopy (collagen). The small white square indicates roughly the region that was analysed by Raman imaging (see Figure 5 and 6, main manuscript). The presence of bacteria in this region was confirmed by fluorescence imaging after staining with an *S. aureus* specific antibody (Figure 6c, main manuscript), validating the results obtained from label-free Raman spectroscopy.

Supplemental Tables

Supplementary Table S1: Overview of tissue slices described in the manuscript

Mouse	Bone	Imaging modality			
		Fluorescence	Raman	H&E	Gram
Infected	Left pelvis*	LF1***			
		LF2 (1.5 mm below LF1)	LR1 (2.9 mm below LF1)		
		LF3 (3 mm below LF1)	LR2 (4.9 mm below LF1)		
		LF4 (4.5 mm below LF1)			
		LF5 (7.5 mm below LF1)			
		LF6 (9 mm below LF1)			
	Right pelvis**	RF1 (35 µm from RG1)		RH1	RG1
		RF2 (35 µm from RG2)		RH2	RG2
Healthy	Right pelvis**			HRH	

*Tissue slices were prepared from cryosections (thickness: 100 µm)

**Tissue slices were prepared from paraffin sections (thickness: 5 µm)

***Slice LF1 is approximately 1.9 mm below the first cut (there were 19 slices before LF1).

Supplementary Table S2: Approximated area of bone tissue.

Tissue section	Area in mm^2 *	Trabecular bone in mm^2 (% of total bone area)	Lesion in mm^2 (% of total bone area)	SAC in mm^2 (% of total bone area)
**Left pelvis**				
LF1	8.0	0.6 (7.5 %)	3.2 (39.6 %)	
LF2	11.4	3.1 (27.0 %)	8.3 (73.0 %)	
LF3	8.2	4.3 (52.8 %)	3.8 (46.0 %)	0.1 (1.2 %)
LF4	4.5	2.1 (47.3 %)	2.4 (52.7 %)	
**Right pelvis**				
RF1	5.4	1.5 (26.9 %)	3.1 (57.7 %)	0.2 (3.5 %)
RF2	4.3	1.4 (33.3 %)	2.2 (51.8 %)	

*Bone area includes the sum of the areas of all bone tissues in the respective tissue slice, i.e. all values given in Supplementary Table S3 and S4, except the muscle tissue regions.

Supplementary Table S3: Approximated area and bacterial region count for muscle tissue, trabecular bone and the lesion with inflamed tissue

tissue slice	Lesion				Trabecular bone				Muscle			
	Total area (μm^2)	Bacterial region count	Bacterial area (μm^2)	relative bacterial occupation in per mille	Total area (μm^2)	Bacterial region count	Bacterial area (μm^2)	relative bacterial occupation in per mille	Total area (μm^2)	Bacterial region count	Bacterial area (μm^2)	relative bacterial occupation in per mille
**LF1***	3189704	328	749	0.235	604601	0	0	0.000	6828744	8	162	0.024
**LF2**	8280480	529	1219	0.147	3069794	2	18	0.006	7658598	10	26	0.003
**LF3****	3786398	212	1922	0.508	4340330	0	0	0.000	8081905	23	486	0.060
**LF4**	2354686	1743	9476	4.024	2109818	13	54	0.025	1967144	5	74	0.038
**mean left §**				**1.2 ± 1.9**				**0.01 ± 0.01**				**0.03±0.02**
**RF1** #**	3142701	1436	2706	0.861	1465646	4	4	0.003	1983558	0	0	0.000
**RF2 #**	2226852	158	214	0.096	1429202	28	36	0.025	1209885	28	33	0.027
**mean right §**				**0.5 ± 0.5**				**0.01 ± 0.01**				**0.01±0.02**

*in this tissue slice, we also found a region rich in chondrocytes and highly vascularized tissue (for size estimation see Supplementary Table S4)
**regions of SACs were excluded from the quantitative analysis of individual bacteria. Total area of SACs in LF3 was 96723 μm^2 and in RF1 was 189282 μm^2
# in these tissue sections we also found bone marrow-like tissue (for size estimation see Supplementary Table S4)
§ mean values are given together with ± standard deviation

Supplementary Table S4: Approximated area and bacterial region count in other tissue types found in specific slices.

tissue slice	Vascularized tissue				Perichondrium				Bone marrow		
	Total area (μm^2)	Bacterial region count	Bacterial area (μm^2)	relative bacterial occupation in per mille	Total area (μm^2)	Bacterial region count	Bacterial area (μm^2)	relative bacterial occupation in per mille	Bacterial region count	Bacterial area (μm^2)	relative bacterial occupation in per mille
**LF1**	3369542	7	108	0.032	881781	2	14	0.016			
**RF1**									12	34	0.052
**RF2**									36	42	0.066
